# Signaling pathways of chronic kidney diseases, implications for therapeutics

**DOI:** 10.1038/s41392-022-01036-5

**Published:** 2022-06-09

**Authors:** Qian Yuan, Ben Tang, Chun Zhang

**Affiliations:** Department of Nephrology, Union Hospital, Tongji Medical College, Huazhong University of Science and Technology, Wuhan, 430022 China

**Keywords:** Kidney diseases, Kidney diseases

## Abstract

Chronic kidney disease (CKD) is a chronic renal dysfunction syndrome that is characterized by nephron loss, inflammation, myofibroblasts activation, and extracellular matrix (ECM) deposition. Lipotoxicity and oxidative stress are the driving force for the loss of nephron including tubules, glomerulus, and endothelium. NLRP3 inflammasome signaling, MAPK signaling, PI3K/Akt signaling, and RAAS signaling involves in lipotoxicity. The upregulated Nox expression and the decreased Nrf2 expression result in oxidative stress directly. The injured renal resident cells release proinflammatory cytokines and chemokines to recruit immune cells such as macrophages from bone marrow. NF-κB signaling, NLRP3 inflammasome signaling, JAK-STAT signaling, Toll-like receptor signaling, and cGAS-STING signaling are major signaling pathways that mediate inflammation in inflammatory cells including immune cells and injured renal resident cells. The inflammatory cells produce and secret a great number of profibrotic cytokines such as TGF-β1, Wnt ligands, and angiotensin II. TGF-β signaling, Wnt signaling, RAAS signaling, and Notch signaling evoke the activation of myofibroblasts and promote the generation of ECM. The potential therapies targeted to these signaling pathways are also introduced here. In this review, we update the key signaling pathways of lipotoxicity, oxidative stress, inflammation, and myofibroblasts activation in kidneys with chronic injury, and the targeted drugs based on the latest studies. Unifying these pathways and the targeted therapies will be instrumental to advance further basic and clinical investigation in CKD.

## Introduction

Chronic kidney disease (CKD) is defined as a decline in renal function [glomerular filtration rate (GFR) <60 mL/min per 1.73 m^2^] or abnormal markers of kidney injury, or both for at least 3 months.^1^ Renal fibrosis is the common pathology of CKD and accelerates CKD progressing into end-stage renal disease (ESRD) that is an end-stage with high mortality.^2^ Nephron loss, inflammation, myofibroblasts activation, and extracellular matrix (ECM) deposition are the main processes of the initial and progression of renal fibrosis.^3,4^ Lipotoxicity and oxidative stress (OS) drive nephron loss.^5^ The pathological analysis of fibrotic kidney biopsy showed cellular lipid accumulated in all kinds of resident renal cells, including tubular epithelial cells (TECs), podocytes, macrophages, endothelial cells, and mesangial cells.^6^ Impaired fatty acid consumption, increased lipid synthesis and uptake, and decreased cholesterol efflux contribute to lipid deposition. The expressions and structures of lipoproteins are also changed in CKD.^7^ Lipotoxicity and modified lipoprotein drive the loss of renal cells by damaging mitochondrial function, promoting OS and inflammation, and even inducing cell death directly.^8^ Damaged mesangial cells and injured podocytes cause glomerulosclerosis and proteinuria. And tubular damage is related to renal interstitial fibrosis. The injured tubules produce and secret a lot of fibrotic factors to activate interstitial myofibroblasts which are ECM generation machines.^9^ The competitive uptake of nutrients and abnormal chemokines secretion of injured resident cells altered the inflammatory microenvironment.^3,10^ For example, damaged TECs produced granulocyte-macrophage colony-stimulating factor persistently that triggered the activation of macrophage, and then the production of MCP-1 that was a ligand of CCR2 expressed by immune cells.^11^ Immune cells such as macrophages, T cells, and B cells are recruited and activated to produce many profibrotic cytokines and growth factors that activate myofibroblasts and promote ECM deposition.^12^ The fibrotic microenvironment triggers renal resident cells such as fibroblasts, pericytes, TECs, and endothelial cells, and bone marrow-derived cells like macrophages, mesenchymal stem cells transdifferentiate into myofibroblasts.^13^ The activation and proliferation of myofibroblasts produce a large amount of ECM. Interstitial ECM expansion accelerates hypoxia and nephron loss. In this review, we discussed the key signaling pathways mediated by lipotoxicity, OS, inflammation, and myofibroblasts activation of CKD. And the effect of inhibitors targeting these pathways on CKD was introduced.

## Signaling pathways related to lipotoxicity in chronic kidney disease

### Normal lipid metabolism

**Fig. 1:**
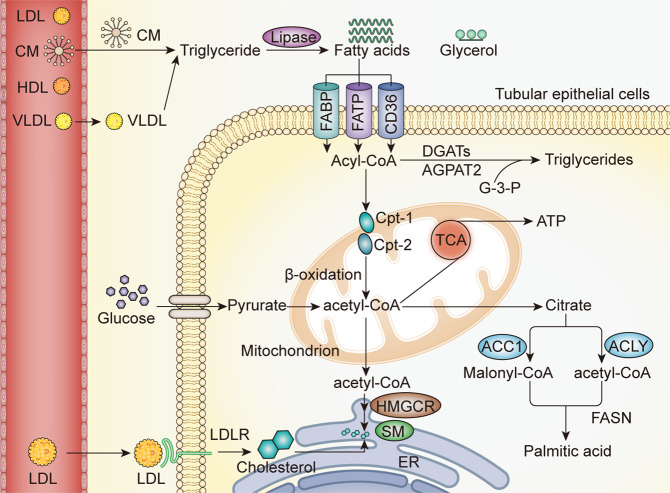
Normal lipid metabolism in tubular epithelial cells (TECs). Triglycerides and cholesterol derived from the diet are mainly carried by CM, VLDL, and LDL. Triglycerides decompose into three FFAs and one glycerol. Fatty acids enter the cells through CD36 or other transporters. Acyl-CoA enters the mitochondrion with the assistance of CPT1 and CPT2. After a complicated process, the acyl-CoA converts into acetyl-CoA, which takes part in the TCA cycle. Acetyl-CoA is also a substrate for palmitic acid and cholesterol synthesis. CM chylomicrons, VLDL very-low-density lipoproteins, LDL low-density lipoproteins, HDL high-density lipoproteins, LDLR low-density lipoproteins receptor, FATP fatty acid transport protein, FABP fatty acid-binding protein, DGAT1 diacylglycerol acyltransferase 1, AGPAT acylglycerolphosphate acyltransferase, HMGCR 3-hydroxy-3-methylglutaryl coenzyme A reductase, SM squalene monooxygenase, ER endoplasmic reticulum, ATP adenosine triphosphate, ACLY ATP citrate lyase, ACC1 acetyl-CoA carboxylase, FASN fatty acid synthase

Lipids are the core energy source and important constructional components of the human body and are essential for normal physiological activities. They are classified into three types: triglycerides, consisting of one glycerol and three fatty acids; phospholipids, which are the major component of the cell membrane; and lipoids, such as cholesterol. Fatty acids are the substrate for adenosine triphosphate (ATP) and some hormones, such as prostaglandin. Fatty acids are categorized into saturated fatty acids (SFAs), monounsaturated fatty acids (MUFAs), and polyunsaturated fatty acids (PUFAs) based on the saturation condition of the carbon chain. Palmitic acid (PA) is a well-known SFA, while omega 3 and omega 6 fatty acids are PUFAs. The intake of SFAs has been demonstrated to be associated with cancer, obesity, myocardial infarction, and insulin resistance by activating the nucleotide-binding oligomerization domain (NOD)-like receptor protein 3 (NLRP3)/apoptosis-associated speck-like protein (ASC) inflammasome.^14–17^ However, PUFAs act oppositely to the SFAs and play a preventive role for these diseases.^18,19^ Phospholipids are a group of lipids containing phosphoric acid. The common shared structure of phospholipids is one hydrophilic head connected with two hydrophobic fatty acyl chains. Phospholipids are divided into sphingolipid and glycerophospholipids that include phosphatidylcholine (PC), phosphatidylethanolamine (PE), phosphatidylserine, phosphatidylinositol, and phosphatidic acid.^20^ The main physiological functions of phospholipids are constituting biological membranes and generating bioactive molecules involved in signal transduction.^21^ Self-synthesis contributes mostly to the cholesterol pool. Cholesterol can be catalyzed into bile acids which are necessary for the digestion of fats, and multiple hormones released by the adrenal cortex and gonads in normal conditions. Cholesterol is also an important component of the cell membrane. A disorder in cholesterol metabolism leads to atherosclerosis, Alzheimer’s disease, and others.^22,23^ FFAs and cholesterol derived from food items are usually carried by lipoproteins. Lipoproteins are classified into chylomicrons (CM), very-low-density lipoproteins (VLDL), low-density lipoproteins (LDL), and high-density lipoproteins (HDL) based on their densities. The density of lipoproteins is determined by the ratio of lipids to apolipoproteins. The detailed compositions of these lipoproteins have been reviewed elsewhere.^24^ Triglycerides are mainly transported by CM and VLDL, while cholesterol is transported by CM, VLDL, and LDL and reverse transported by HDL (Fig. 1).

The metabolism of triglycerides and cholesterol participates in different kinds of important physiological activities in the human body. They are obtained from the diet and then transported to the blood circulation in the form of lipoproteins, which consist of apolipoprotein and lipids. Following hydrolysis by lipase, the fatty acids and glycerol are released from triglycerides. FFAs can be utilized by TECs as their main fuel through CD36 or other transport proteins, such as fatty acid transport protein (FATP) and fatty acid-binding protein.^25,26^ FFAs are then activated to acyl-CoA by synthetases to make them permeable for mitochondrion. Acyl-CoA passes through the outer membrane of a mitochondrion with the assistance of carnitine palmitoyltransferase-1 (CPT1) and enters through the inner membrane by the transportation of CPT2^27^ (Fig. 1). The acyl-CoA is converted into acetyl-CoA in the mitochondrial matrix through a series of dehydrogenation and thiolysis reactions. Finally, the acetyl-CoA participants in the tricarboxylic acid cycle (TCA). Nicotinamide adenine dinucleotide and flavin adenine dinucleotide, produced through β-oxidation, provide protons in the process of ATP generation in mitochondrion. Excessive FFAs are esterified into acyl-CoA, which assembles in the glycerol-3-phosphate backbone and is stored as triacylglycerol. Furthermore, lipids can also be derived from glucose metabolism, which is termed as de novo lipogenesis. Glucose produces citrate through the TCA cycle, which is converted into PA through the successive catalyzation of ATP citrate lyase or acetyl-CoA carboxylase (ACC1) and fatty acid synthase (FASN)^28^ (Fig. 1). Acetyl-CoA, a substrate in the TCA cycle, is also used in cholesterol synthesis, which occurs in almost all mammalian cells, except hepatocytes, adrenal cells, and gonadal cells.^29^ In the action of a series of enzymes, including two rate-limiting enzymes 3-hydroxy-3-methylglutaryl coenzyme A reductase and squalene monooxygenase, the acetyl-CoA is converted into cholesterol around the endoplasmic reticulum (ER)^30^ (Fig. 1). To balance the cellular cholesterol level, ATP-binding cassette (ABC) transporter subfamily A member 1 (ABCA1) and ABC subfamily G (ABCG) members 1, 5, and 8 are responsible for the cholesterol efflux.^29^ The metabolites of glucose, lipid, and amino acid metabolism such as ethanolamine (EA), and choline are substrates for phospholipids synthesis. EA and choline are catalyzed into CDP-EA, and CDP-choline by phosphoethanolamine cytidylyltransferase and phosphocholine cytidylyltransferase respectively.^31^ And CDP-EA, CDP-choline act with diglyceride to form PC and PE respectively. The catabolism of phospholipids is mediated by phospholipase including A1, A2, B1, B2, C, and D. The phospholipase A2 receptor (PLA2R) was revealed as an antigenic target in autoimmune adult membranous nephropathy and anti-PLA2R antibody was regarded as a specific biomarker of membranous nephropathy.^32^

### Disorder of lipid metabolism in CKD

The renal lipid deposition phenomenon has been observed in many animal studies (Table 1). Lipid drops are present in almost all the renal parenchyma cells, suffering from an injury.^6^ Using large genome-wide transcriptome analysis, an elegant study in 2015 revealed that inflammation and metabolism were the topmost dysregulated pathways in human diseased kidneys.^33^ Kang et al. found low levels of key enzymes and regulators related to fatty acid oxidation (FAO) and accumulation of lipids in TECs in both the human and mouse fibrotic kidneys. The major fatty acid subtypes, which were deposited in the kidney of the mice with the TEC conditional overexpression of long-chain fatty acid transporter CD36, were stearic, palmitic, linoleic, and docosahexaenoic acids (DHA).^33^ The deposition of lipids in TECs was also found in angiotensin II-infused rats, high-fat diet (HFD) mice, and cisplatin-induced nephrotoxicity models.^34–36^ More importantly, this phenomenon was also found in human studies. For example, lipids accumulated in mesangial cells, podocytes, and TECs of patients with obesity-related kidney disease and diabetic nephropathy (DN).^37,38^

**Table 1:** Lipid deposition of different renal cells in CKD animals

Renal cell type	Animal model	Treatment in vitro	Ref.
Tubular epithelial cells	Folic acid nephropathy	TGF-beta1	^33^
Tubular epithelial and vascular wall cells	Angiotensin II-infused rat	–	^34^
Podocyte	STZ-induced diabetic nephropathy mice and db/db mice	High glucose	^68,74^
Tubular epithelial cells, mesangial cells	High-fat diet mice	Palmitic acid	^49,94,158^
Podocyte	Podocyte-specific Abca1 knockout mice	–	^79^
Podocyte	TNF-injected mice	TNF	^80^
Tubular epithelial cells	Cisplatin-induced nephrotoxicity, 5⁄6 nephrectomy	–	^36,400^
Mesangial cells, tubular epithelial cells	Aging mice or rat	TGF-beta1	^51,67^
Tubular epithelial cells	Unilateral ischemia-reperfusion nephropathy	–	^52^
Endothelial cells	ApoE-deficient mice with high-phosphate diet	–	^401^
Macrophage	Uninephrectomy of high-fat diet-treated ApoE-deficient mice	–	^402^
Endothelial cells	apoE-/- diabetic mice	High glucose	^403^

Other than lipid accumulation in kidney tissues, CKD patients always have dyslipidemia, which is characterized by high levels of serum triglycerides, LDL-cholesterol (LDL-C), and low HDL-cholesterol (HDL-C) levels. The high levels of LDL-C and oxidative-LDL (ox-LDL) are independent risk factors for CVD, which is the leading cause of death among patients with eGFR <60 mL/min per 1.73 m^2^.^39,40^ Dyslipidemia in nephrotic syndrome (NS) is characterized by high LDL and VLDL levels and normal HDL levels, which are different from uremia dyslipidemia.^41^ Despite the comparable level of HDL in NS patients and healthy people, the reverse cholesterol transportation function of HDL may be impaired.^42^

### Key signaling pathways of renal lipid deposition and altered lipoprotein metabolism

**Fig. 2:**
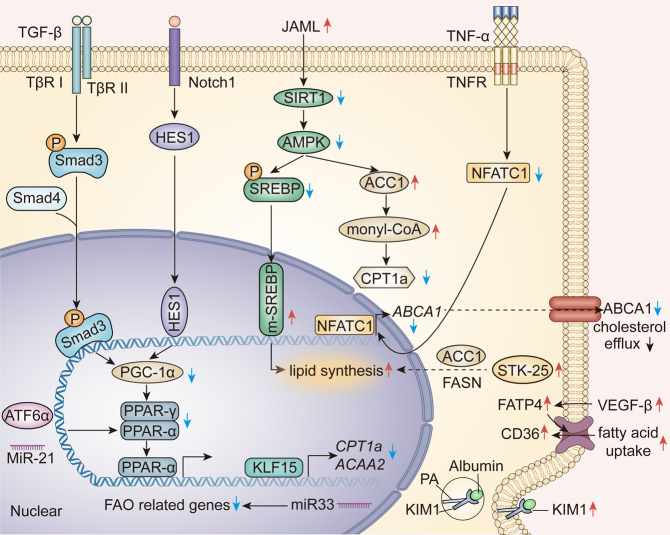
Mechanisms of renal lipid deposition. TGF-β/Smad3 signaling and Notch1/HES1 signaling mediate the downregulation of PGC-1α, which is a key regulator of the PPAR family and decreases the expression of FAO-related genes. ATF6α and miR-21 transcriptionally decrease the expression levels of PPAR-α. The miR-33 and loss of KLF15 downregulate the expression of FAO-related genes directly. The low level of AMPK activates ACC1, which promotes the formation of monyl-CoA and decreases the expression of CPT1a. The activation of JAML inhibits the SITR1-AMPK axis, resulting in SREBP1 upregulation and lipid synthesis. STK-25 is also involved in lipid synthesis. TNF-α inhibits the nuclear translocation of NFATC1 and blocks the cholesterol efflux by ABCA1. VEGF-β, CD36, and KIM-1 mediate the ectopic uptake of fatty acids in CKD. TGF-β transforming growth factor-β, PPAR peroxisome proliferator-activated receptor, PGC-1α PPARγ coactivator 1α, FAO fatty acid oxidation, ATF6α activating transcription factor 6α, KLF15 Krüppel-like factor 15, AMPK AMP-activated protein kinase, ACC1 acetyl-CoA carboxylase 1, CPT1a carnitine palmitoyltransferase 1a, JAML junctional adhesion molecule-like protein, SREBP element-binding protein, STK-25 serine/threonine-protein kinase 25, TNF-α tumor necrosis factor-α, NFATC1 nuclear factor of activated T cell 1, ABCA1 ATP-binding cassette transporter A1, VEGF-β vascular endothelial growth factor-β, KIM-1 kidney injury molecule-1

Normal cellular lipid level is maintained by the balance of lipid synthesis, FAO, lipid uptake, and export. Abnormality in any of the normal processes destroys the balance, leading to lipid deposition (Fig. 2). The characteristics of lipid metabolism in CKD are a disturbance in FAO, increased lipid synthesis, enhanced lipid uptake, and obstructed lipid efflux. The levels and structures of lipoproteins are altered in CKD due to proteinuria and the uremic microenvironment.

#### Destruction of FAO

The disturbance in the main consumption way of fatty acids, β-oxidation, causes lipid accumulation and direct renal damage. Transcriptomic sequencing analysis showed the downregulation of FAO-related genes, including *CPT1a*, acyl-coenzyme A oxidase 1 (*ACOX1*), *ACOX2*, peroxisome proliferator-activated receptor α (*PPARα*), and *PPARγ* coactivator 1α (*PPARGC1A*, coding PGC-1α) in the human and mice fibrotic kidney.^33,43^

The mitochondrion is an important organelle, where FAO is carried out and its injury is always associated with FAO disturbance. Transforming growth factor-β (TGF-β) that is the master driving factor of renal fibrosis has been demonstrated to damage FAO by decreasing the expression of mitochondrial biogenesis determinant gene, PGC-1α in the Smad3-dependent pathway.^33,44^ TECs specifically overexpressing PGC-1α normalized transcript levels of Cpt and other FAO enzymes after TGF-β treatment.^33^ And PGC-1α modulation correlates directly to altered lipid levels. Hepatic PGC-1α overexpression reduced triacylglycerol (TAG) storage and secretion, resulting in decreased plasma TAG levels.^45^ Transgenic mice with tubule-specific overexpression of intracellular Notch1 domain showed low renal mitochondrial contents and defective FAO, which were reversed by the overexpression of PGC-1α.^46^ Mechanistically, the transcription factor Hes1 regulated by Notch signaling decreased the expression of PGC-1α by binding to its promoter.^46^

PPAR family, especially the PPARα, is known as the master regulator of FAO since the β-oxidation level of long-chain fatty acid in PPARα knockout mice decreased by 70% compared to wild-type mice.^47,48^ PPAR agonists, such as fenofibrate, play a renal protective role in a series of kidney diseases, including ischemia-reperfusion injury, DN, HFD-induced nephropathy, and hypertensive nephropathy.^49,50^ The kidneys of aged PPARα-knockout mice showed renal lipid accumulation and decreased expression levels of FAO-related genes, such as *CPT1a*, *ACOX1*, medium-chain acyl-CoA dehydrogenase (Mcad), and Acyl-CoA dehydrogenase family member 9 (ACad9).^51^ Negative regulators of PPARα, miR-21, and transcription factor ATF6α led to successive lipid deposition, mitochondrial dysfunction, and renal fibrosis.^52^ Another member of the PPAR family, PPAR-γ, which is highly expressed in the adipocytes, is a key regulator of adipogenesis and lipid metabolism.^53,54^ Decreased expression of PPAR-γ could alter the microenvironment of adipose tissues, which was characterized by white adipocyte hypertrophy and increased inflammation.^55^ These adipocytes release FFAs and metabolites, including inflammatory factors, such as interleukin-6 (IL-6), and tumor necrosis factor (TNF-α), and hormonal factors, such as leptin, angiotensin II, which mediate kidney injury.^56^ In other tissues, like the liver, kidney, and heart, PPAR-γ is also expressed at a low level. PPAR-γ regulates many lipid transport- and metabolism-related genes, such as acyl-CoA synthase, FATP, CD36, and lipoprotein lipase (LPL).^57^ Global PPAR-γ knockout mice showed renal hypertrophy and increased albuminuria in 3rd week. In the 7th week, the mice showed decreased creatinine clearance and developed type 2 diabetes, while the 52 weeks old mice showed server inflammation and renal fibrosis.^58^ However, to the best of our knowledge, studies demonstrating the correlation between PPAR-γ and renal lipid deposition are limited.

AMP-activated protein kinase (AMPK), a low-energy sensor, is known to be inactive in CKD.^59^ The low level of AMPK could activate ACC2 and promote the formation of malonyl-CoA, leading to the downregulation of CPT1a.^60^ The upregulation of AMPK by metformin treatment phosphorylated ACC1 at Ser79 and improved FAO.^61^ Furthermore, AMPK could also induce mitochondrial biogenesis by promoting the expression of PGC-1α, thereby facilitating FAO.^62^ The loss of miR-33 could protect mice from unilateral ureteral obstruction (UUO) and folic acid-induced renal fibrosis by recovering the FAO-related factors.^63^ A recent study revealed another transcriptional factor, Krüppel-like factor 15 (KLF15), which was highly expressed in the normal proximal TECs and could mediate the expression of CPT1a and acetyl-coenzyme A acyltransferase 2 (ACAA2).^64^ In the kidney of UUO mice, diminishing KLF15 transcriptionally downregulated CPT1a and ACAA2, resulting in compromised FAO and renal fibrosis.^64^

#### Lipogenesis

Fat tissues and the liver are the main sites for the synthesis of triglyceride and cholesterol, respectively. However, when the kidney suffers from an injury, lipid metabolism is changed. Sterol regulatory element-binding protein (SREBP) is an important transcription factor, which regulates the biosynthesis of cholesterol, fatty acids, triacylglycerol, and phospholipids.^29,65^ SREBP1a overexpression transgenic mice showed increased levels of FASN and ACC, resulting in glomerular triglyceride deposition, mesangial expansion, glomerulosclerosis, and proteinuria.^66^ The upregulation of SREBP in aged kidneys and DN resulted in lipid accumulation in the glomerulus.^66,67^ Fu et al. revealed that the junctional adhesion molecule-like protein expressed by the damaged podocytes causes an increased lipid synthesis through the SIRT1-AMPK-SREBP1 axis.^68^ Besides lipotoxicity, SREBP directly mediates renal fibrosis by increasing the expression levels of fibrotic factors, such as TGF-β1, plasminogen activator inhibitor-1 (PAI-1), and COL6A1.^69^ Transcription factor farnesoid X receptor (FXR), a bile acid-activated nuclear hormone receptor, protects kidneys by inhibiting the SREBP1.^70^ The mRNA and protein levels of FXR were found to be decreased in human DN and obesity-induced nephropathy.^71^ Serine/threonine-protein kinase 25 promotes lipid deposition and renal injury by increasing the expression levels of fatty acid de novo synthesis genes, such as FASN and ACC1.^72^

#### Enhanced uptake and disturbed export of lipid

CD36 is a major transporter for the uptake of FFAs and is widely expressed in the renal parenchymal cells, including TECs, macrophages, podocytes, mesangial cells, and endothelial cells.^26^ Immunohistochemical staining of kidney biopsy from the CKD patients showed that the upregulation of CD36 was correlated with lipid deposition.^38,73^ The tubule-specific overexpression of CD36 in transgenic mice exhibited excessive lipid amassing.^33^ Falkevall et al. demonstrated that the increased level of vascular endothelial growth factor- β (VEGF-β) in the podocytes of DN mice could promote the uptake of FFAs by upregulating the FATP4.^74^ FFAs bound albumin in the urine can be reabsorbed by TECs. Transmembrane fatty acid transporter-2 (FATP-2) exclusively expressed on the apical side but not on the basolateral side of tubules mediates the uptake of fatty acid-bound albumin.^75^ Besides transporting proteins, the TECs can also uptake the fatty acids-bound albumin through endocytosis.^27^ Kidney injury molecule (KIM)-1 is a well-known tubular injury factor for both acute kidney injury (AKI) and CKD. However, a recent study by Yutaro et al. showed that the increased level of KIM-1 in DN could mediate the endocytosis of PA-bound albumin, leading to tubular DNA damage, cell cycle arrest, inflammation, and fibrosis.^76^

ABCA1 is a transporter, which mediates the cellular cholesterol and phospholipid efflux in an ATP-dependent pathway.^77^ A large-scale transcriptional analysis of ABCA1 in the DN patients revealed a decrease in its expression in the early stage of DN.^78^ Podocytes with the ABCA1 gene knocked out aggravated the podocyte injury in ob/ob mice by inducing the accumulation of cardiolipin and mitochondrial dysfunction. And ABCA1 inducer A30 ameliorated the glomerular sclerosis and proteinuria in db/db mice.^79^ TNF-α treatment downregulated the expression of nuclear factor of activated T cells 1/ABCA1 axis, leading to the cholesterol-induced podocytes injury.^80^

#### Lipoprotein metabolism disorder

The metabolism, structure, and molecular composition of lipoproteins in CKD patients differ from healthy people. CKD patients are always accompanied by low levels of HDL and high levels of LDL. The main apolipoprotein of HDL is ApoA-I. However, the expression of ApoA-I in CKD patients and animals is decreased. The possible reasons are reduced biosynthesis and increased urine excretion.^81^ The mature process of HDL needs lecithin-cholesterol acyltransferase (LCAT) to catalyze the esterification of the cholesterol. But, the expression of LCAT was downregulated and the catalyzed activity was impaired in patients with CKD. This impaired the maturation of HDL.^82^ Except for the decreased levels, the composition of HDL has been changed in CKD patients. Uremic solute, symmetric dimethylarginine (SDMA) accumulates in CKD patients due to the decreased urine excretion and forms complex with HDL. SDMA enriched HDL was no longer beneficial for cardiovascular diseases but caused endothelial cell dysfunction.^83^ Acute phase reaction protein, serum amyloid A (SAA1) was significantly elevated in the serum of CKD patients and accelerated renal interstitial fibrosis by inducing ER stress.^84^ SAA1 can replace ApoA-I and become the main apolipoprotein of HDL, leading to the proinflammatory effect and reduced cholesterol efflux capacity.^24^ The ratio of ApoC-III was elevated in CKD patients and resulted in HDL dysfunction.^85,86^ The uremia microenvironment promotes post-translational modifications such as carbamylation and oxidation of lipoproteins. Carbamylation modification of lysine residues of ApoA-I rendered HDL dysfunction and was associated with increased mortality outcomes in patients with type 2 diabetes.^87^ The disordered metabolism of LDL in CKD includes increased levels and post-translational modifications. Reduced LDL catabolism and the associated increased LDL residence time contribute to the increased LDL levels and enhance post-translational modifications such as oxidation, carbamylation, and nitration of LDL.^39^ Oxidized LDL (ox-LDL) impaired the binding ability with LDL receptors, resulting in decreased clearance in the liver. And hepatic LDL receptor dysfunction is also one of the mechanisms of reduced LDL catabolism.^88^ Increased scavenger receptors, such as lectin-like-oxidized LDL receptor 1 (LOX1), CD36, scavenger receptor-A1 (SR-A1), and SR-A2 in CKD mediate the increased uptake of ox-LDL, leading to the activation of inflammatory signaling pathways.^89,90^ Increased ApoC-III levels and reduced ApoC-II to ApoC-III ratio block the activity of LPL and hepatic triglyceride lipase that mediate the conversion of VLDL to intermediate-density lipoprotein (IDL) and IDL to LDL, respectively. This process increases the production of LDL.^24^

### Signal pathways mediated lipid nephrotoxicity

**Fig. 3:**
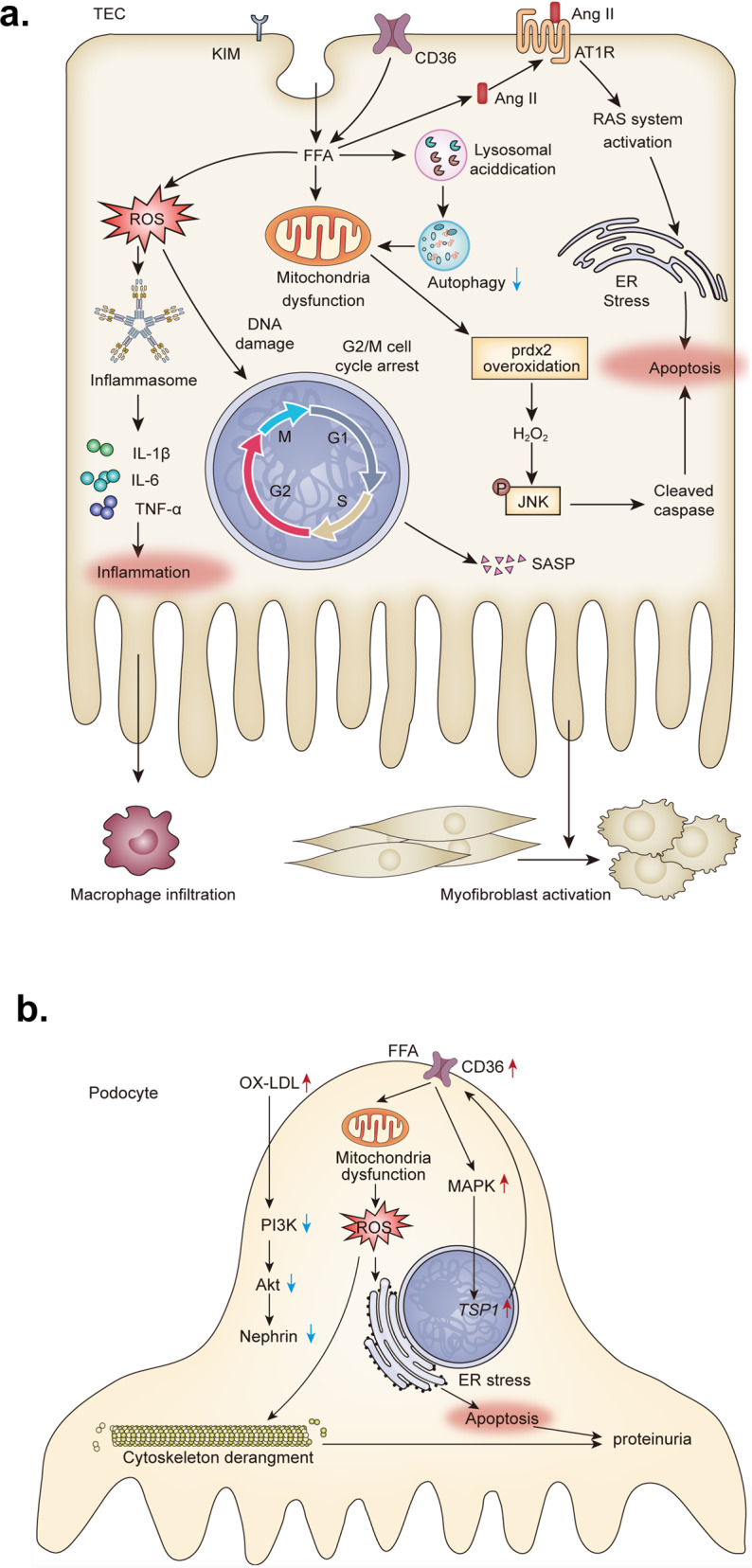
Lipotoxicity of tubular epithelial cells and podocytes. **a** In TEC, the accumulation of FFAs triggers mitochondrial dysfunction directly or by decreasing autophagy. Hydrogen peroxide produced by injured mitochondria activates JNK and cleaves caspase-3, leading to the apoptosis of TEC. Apoptosis caused by FFA is also mediated by the activation of the RAAS system. FFAs induce the production of ROS, which activates inflammasome and damages DNA. DNA damage inhibits the cell cycle and causes cell senescence. The senescent TEC releases cytokines and chemokines, which recruit immune cells, such as macrophages and fibrotic factors, which activate fibroblasts. **b** In podocytes, the ox-LDL induces loss of nephrin through PI3K/Akt signaling. FFAs cause mitochondrial dysfunction, oxidative stress, and ER stress, resulting in the derangement of cytoskeleton and apoptosis of podocytes. FFA-TSP1 vicious cycle aggravates the lipid damage. TEC tubular epithelial cell, FFA free fatty acid, JNK c-Jun N-terminal kinase, Ang II angiotensin II, RAAS renin-angiotensin system, SASP senescence-associated secretory phenotype, KIM-1 kidney injury molecule-1, ROS reactive oxygen species, PI3K phosphoinositide 3-kinase, MAPK mitogen-activated protein kinases, ER endoplasmic reticulum, TSP1 thrombospondin1

Lipids are usually stored in fat tissues in the form of lipid drops. When lipids accumulate in other tissues and cause damaging effects, the phenomenon is known as lipotoxicity.^8^ Lipid nephrotoxicity was first proposed by Moorhead et al. in 1982.^91^ The deposition of lipids, including FFAs, cholesterol, and phospholipids, induces mitochondrial dysfunction, OS, inflammation, ER stress, and even apoptosis of renal cells (Fig. 3). Lipid accumulation is associated with increased proteinuria, reduced eGFR, and renal fibrosis. Lipid-lowering drugs, such as statins, attenuate the progression of CKD and reduce proteinuria.^92^ Altered lipoproteins promote CKD progression by triggering the OS and inflammation process.

#### Tubular epithelial cells

Renal tubule cells are the most abundant cells in the kidney and maintain the functions of renal filtration and reabsorption. Tubular injury or apoptosis accelerates the CKD progression by the loss of nephron, thereby aggravating the inflammation and fibrosis. KIM-1 mediates the endocytosis of PA-bound albumin, leading to reduced mitochondrial transmembrane potential and mitophagy.^76^ Mitochondrial dysfunction and lipid overload increase the production of reactive oxygen species (ROS), which triggers the activation of inflammasome and secretion of IL-1β, IL-6, and TNF-α. ROS also induces DNA damage and G2/M cell cycle arrest in TECs, resulting in the cell senescence and partial epithelial to mesenchymal transition (partial EMT).^5,76^ Damaged tubules exhibit senescence-associated secretory phenotype that is characterized by secreting profibrotic factors, such as TGF-β1, Wnt, and sonic hedgehog. These factors promote the activation and proliferation of fibroblast and the production of ECM in renal interstitial fibrosis (Fig. 3).^2^ Another study revealed that the increase of fatty acid-bound albumin but not the albumin in TECs impaired the mitochondrial function and over oxidated the peroxiredoxin 2 (PRDX2). Then, the increased peroxide levels activated the p- c-Jun N-terminal kinase (JNK) /caspase-3 apoptotic pathway, resulting in the apoptosis of cells.^93^ HFD induces tubular lysosomal acidification and decreases autophagy, thereby exaggerating the mitochondrial dysfunction and inflammasome activation.^94^ These studies demonstrate that mitochondrial dysfunction plays a key role in the FFA-induced damage to the TECs. The mechanism of mitochondrial damage caused by lipids has not been clarified yet. The mitochondrial injury might be induced by mitochondrial FFA or ox-LDL overload due to the presence of their receptor CD36 on the mitochondrial membrane.^26^ PA treatment also induced the expression of angiotensin II. And the activation of the renin-angiotensin system (RAAS) caused ER stress.^95^ Although much evidence showed FFA impaired TECs directly, Kang et al. demonstrated that the transgenic mice with tubular-specific overexpression of CD36 showed tubular lipid accumulation but not renal fibrosis. Transgenic mice showed higher levels of ECM-related genes at 20 weeks old, but the difference was insignificant as compared to the wild-type mice.^33^ It was suspected that obvious statistical differences might be observed in elder mice.

As FAO is the preferred energy source of TECs, defective FAO causes tubular damage naturally.^33^ CPT1a is a key rate-limiting enzyme for the β-oxidation of FFAs. Tubular-specific knockout of *CPT1a* gene in mice showed elevated levels of serum creatine, proteinuria, tubular damage, and tubular interstitial fibrosis at the age of 18–20 months.^96^ Among the CKD patients, renal CPT1a level was negatively correlated with fibrosis score.^97^ Furthermore, lower oxygen consumption rates and the impaired mitochondrial complex I and V of primary TECs isolated from the 9-month-aged *CPT1a* knocked out transgenic mice indicated that the disturbance in β-oxidation caused mitochondrial damage and then renal injury.^96^ And tubular-specific overexpression CPT1a mice showed attenuated renal fibrosis, inflammation, and tubular injury when suffering from UUO, folic acid nephropathy, and adenine-induced nephrotoxicity compared to wild-type mice.^97^ CPT1a inhibitor etomoxir could aggravate folic acid nephropathy by inhibiting the FAO.^33^

#### Podocytes

The podocyte foot processes effacement and podocyte apoptosis are the leading cause of proteinuria and subsequent glomerular sclerosis. Clinical and animal studies have revealed lipid biology as a key determinant of podocyte function.^98^ As compared to TECs, the podocytes are highly susceptible to saturated FFAs, but not to monounsaturated FFAs.^99^ PA causes mitochondrial dysfunction and release of calcium ions from ER, leading to the derangement of podocyte cytoskeleton and apoptosis.^99–101^ The increased uptake of FFA mediated by CD36 induces the upregulation of thrombospondin1 (TSP1) which is also a ligand of CD36 in a p38 mitogen-activated protein kinase (MAPK) signaling-dependent way. And TSP1 treatment induces podocyte apoptosis.^102^ The TSP1-deficient mice showed the attenuation of obesity, diabetes, and adriamycin-associated podocytes apoptosis.^102–104^ The vicious cycle of FFA and TSP1 is an important mechanism of podocyte injury (Fig. 3). PA also inhibits the phosphorylation of insulin receptors, such as IRS1 and PKB, and blocks insulin-stimulated glucose uptake in human podocytes.^105^ The insulin resistance of podocytes is associated with podocytes damage and loss of podocytes foot processes.^106^ Although fatty acids are probably not the preferred energy source of podocytes, FAO disturbance causes podocytes’ injury. The stimulation of FAO by adiponectin, which is an activator of AMPK or aminoimidazole-4-carboxamide-1-D-ribofuranoside (AICAR), protects the podocytes from PA injury, while the CPT1a inhibitor augments PA toxicity and abolishes the beneficial effects of AICAR.^60^ Besides FFAs, other lipids damage the podocytes too. The peroxidation of mitochondrial cardiolipin mediated by ABCA1 promotes podocytes injury in DN.^79^ The ox-LDL inhibits the phosphorylation of phosphatidylinositol 3-kinase (PI3K) and Akt, leading to the redistribution and loss of nephrin.^107^ The G1 and G2 renal-risk variants of the apolipoprotein L1 gene (APOL1) have caused glomerulosclerosis in many African Americans.^108^

#### Mesangial cells

Mesangial cells in normal glomerulus secrete minimal matrix to support capillary loops. However, the FFAs and ox-LDL cause the direct injury of mesangial cells. PA treatment-induced monocyte chemoattractant protein-1 (MCP-1) expression of mesangial cells by inhibiting the phosphorylation of AMPK.^109^ Increased MCP-1 recruited macrophages, dendritic cells, and T cells from bone marrow by arising the expression of intercellular adhesion molecule 1 (ICAM-1) and enhancing monocyte adhesion, resulting in severe inflammation of CKD.^11,110^ Furthermore, PA could also increase the expression of protein arginine methyltransferase 1 and induce mesangial apoptosis by activating the ER stress.^111^ The inhibition of FAO by etomoxir increased the PA-induced mesangial apoptosis.^112^ The glycation of apolipoprotein B reduces the binding affinity of LDL to LDL receptors in mesangial cells, leading to the trapping of LDL in the ECM.^113^ The uremic microenvironment modifies LDL into ox-LDL. The uptake of ox-LDL mediated by scavenger receptors activates the PAI-1 transcription through TGF-β signaling.^114^ The increased uptake of cholesterol and triglycerides transforms mesangial cells into foam cells.^37,115^ Injured mesangial cells lose contractile function and produce excessive ECM, leading to glomerular sclerosis.

#### Macrophages and endothelial cells

A small number of macrophages are renal resident cells. However, the lipotoxicity of other renal cells promotes the release of chemokines and cytokines, which recruit and activate macrophages from blood circulation. Activated macrophages produce much more inflammatory cytokines, ROS, and nitric oxide (NO), leading to further renal damage. The upregulated expression of ICAM-1 in glomerular endothelial cells by ox-LDL treatment increases the adhesion and infiltration of monocytes within the glomerulus.^116^ Lipid also acts directly on macrophages and mediates the progression of CKD and its cardiovascular complications. The uptake of ox-LDL and modified lipoproteins by macrophages aggravate glomerulosclerosis and increase the risk of atherosclerosis.^117^ Ox-LDL stimulates the activation of caspase-3 and promotes the apoptosis of macrophages in both the CD36 and Toll-like receptors (TLR2)-dependent pathways, leading to plaque necrosis in advanced atherosclerosis.^118,119^ And atherosclerosis aggravation can elevate the mortality of CKD. The knockout of CD36 in macrophages ameliorated renal lipid deposition, followed by preserved renal function and reduced fibrosis.^120^

The intact endothelial cells in tubular interstitial space guarantee the transportation of serum oxygen. Besides oxygen, serum lipids are also carried by vessels. VEGF-β specifically controls the FFA uptake of endothelial cells by upregulating vascular FATPs.^121^ Therefore, the expression of VEGFs determines the tissue distribution of serum lipids. Ablation of VEGF-β reduced renal lipid accumulation in db/db mice.^74^

### Therapeutic opportunities of CKD based on lipid metabolism

The above-mentioned studies showed a strong association between lipotoxicity and CKD progression. Therefore, the lipid-lowering strategy might be a potential therapy for CKD. The possible lipid-lowering drugs and their effects on preserving eGFR and reducing proteinuria and the risk of ESRD are summarized in Table 2. Alternative drugs for reducing the lipid accumulation of DN have been reviewed elsewhere.^8^

**Table 2:** Potential CKD drugs based on lipid metabolism

Target	Drug	Animal model/human disease	Results	Ref.
HMG-CoA inhibitor	Simvastatin	CKD patients without dialysis	Reduced the risk of atherosclerotic events, nonhemorrhagic stroke, and arterial revascularization	^123^
HMG-CoA inhibitor	Atorvastatin	CKD patients	Modest beneficial effect on eGFR; decreased urine protein: creatinine ratio	^127^
HMG-CoA inhibitor	Simvastatin	SHR feed with HFD	Reduced glomerulosclerosis and proteinuria	^131^
HMG-CoA inhibitor	Lovastatin	PA nephrosis	Reduced inflammation in glomeruli	^132^
PPARα agonist	Fenofibrate	Elder type 2 diabetic patients with moderate eGFR (≥30 mL/min/1.73 m^2^)	Reduced total cardiovascular events and preserved renal function without adverse effect	^135^
PPARα agonist	Fenofibrate	HFD mice, FAN model	Ameliorated glomerular injury, inflammation, and renal fibrosis	^33,49^
PPARα agonist	Clofibric acid	5/6 nephrectomy	Improve cardiac function and decrease urine albumin excretion but not serum creatine	^137,138^
PPARγ agonist	Pioglitazone	5/6 nephrectomy and aging rats	Reduced proteinuria, improved GFR, decreased sclerosis	^142,143^
PPARγ agonist	Rosiglitazone	Type 2 diabetic patients	Decreased ACR compared with metformin, preserved eGFR compared with glyburide	^140^
CD36 antagonist	5A	UUO	Attenuated inflammation and renal fibrosis	^144^
Mitochondrial-targeted antioxidant peptide	SS-31	HFD mice	Abolished lipotoxicity, loss of podocytes and endothelial cells, glomerulosclerosis, and inflammation	^146^
Mitochondrial-targeted antioxidant drug	MitoQ	db/db mice	Prevented glomerular hypertrophy and mesangial expansion	^148^
FXR agonist	GW4064	db/db mice	Decreased proteinuria, glomerulosclerosis, and interstitial fibrosis	^156^
FXR agonist	INT-747	STZ-induced DN	Modulated lipid accumulation and reduced proteinuria, glomerulosclerosis, and tubulointerstitial fibrosis	^70^
TGR5 agonist	INT-777	Obesity-associated nephropathy and DN	Decreased proteinuria, podocyte injury, mesangial expansion, fibrosis, and CD68 macrophage infiltration	^157^
Dual FXR/TGR5 agonist	INT-767	Obesity-associated nephropathy and DN	Improved proteinuria and prevented podocyte injury, mesangial expansion, and tubulointerstitial fibrosis	^71^
AMPK agonist	A-769662	5/6 nephrectomy mice fed a high-protein diet	Ameliorated renal fibrosis	^59^
AMPK agonist	AICAR	HFD mice	Improved lipid accumulation, mitochondrial dysfunction, lysosomal dysfunction, and renal fibrosis	^158^
ABCA1 inducer	A30	DN	Ameliorated podocyte injury	^79^

*HMG-CoA* 3-hydroxy-3-methyl-glutaryl-coenzyme A, *SHR* spontaneously hypertensive rats, *HFD* high-fat diet, *PA* puromycin aminonucleoside, *PPAR* peroxisome proliferator-activated receptors, *FAN* folic acid nephropathy, *UUO* unilateral ureteral obstruction, *ACR* albumin/creatinine ratio, *STZ* streptozotocin, *TGR5* G protein–coupled receptor 5, *AMPK* AMP-activated protein kinase, *ABCA* ATP-binding cassette (ABC) transporter subfamily A member

#### Statin

Statin is the inhibitor of 3-hydroxy-3-methyl-glutaryl-coenzyme A (HMG-CoA) that is a rate-limiting enzyme of cholesterol synthesis and is regarded as the primary lipid-lowering drug for CKD patients. SHARP (Study of Heart and Renal Protection) studies revealed that the CKD patients, who did not undergo dialysis and were treated with simvastatin (20 mg daily) and ezetimibe (10 mg daily), showed a reduction in major atherosclerotic events, nonhemorrhagic stroke, and arterial revascularization by 17%, 25%, and 21%, respectively, as compared to the placebo group. However, for the CKD patients, receiving either hemodialysis or peritoneal dialysis (PD), the statin treatment had little or no beneficial effect on CVD.^122,123^ A similar conclusion was also revealed by the clinical trials of 4D (German Diabetes Dialysis Study) and AURORA (A Study to Evaluate the Use of Rosuvastatin in Subjects on Regular Hemodialysis: An Assessment of Survival and Cardiovascular Events).^124,125^ Besides reducing the CVD risk, statins, such as atorvastatin, also showed a modest beneficial effect on eGFR.^126^ Prospective Evaluation of Proteinuria and Renal Function in Diabetic Patients with Progressive Renal Disease (PLANET I) trial indicated that the treatment of 80-mg atorvastatin decreased urine protein to creatinine ratio by 13% after 52 weeks as compared to baseline.^127^ Statins play a protective role for both cardiovascular and renal diseases in CKD patients. The molecular mechanisms for the renal protective role of statins include not only the renal advantageous effect of reducing lipid deposition, but also include its direct anti-inflammatory, antioxidant, pro-apoptotic, and antifibrotic effects on renal parenchymal cells.^128,129^ Simvastatin restricted the proliferation of mesangial cells by reducing the expression of cell cycle-dependent kinase and reduced proteinuria of spontaneously hypertensive rats fed with HFD by protecting endothelial cells from OS.^130,131^ Lovastatin reduces the expression of MCP-1 and macrophage infiltration in the glomerulus.^132^ Different statins can inhibit podocyte injury and apoptosis by activating the PI3K/AKT-signaling pathway.^107^ It is worth noting that the high dose of lovastatin treatment might induce apoptosis and inhibit the proliferation of TECs.^133,134^

#### PPAR agonist

The transcription factor, PPAR-α, is primarily involved in the regulation of FAO. Fenofibrate is a selective agonist of PPAR-α. Fenofibrate prevented the FFA-induced lipid accumulation and OS of mesangial cells and TECs in vitro.^49^ Consistently, fenofibrate ameliorated glomerular injury, inflammation, and renal fibrosis in vivo.^33,49^ Fenofibrate Intervention and Event Lowering in Diabetes (FIELD) study enrolled the elder type 2 diabetic patients with moderate eGFR (≥30 mL/min/1.73 m^2^) to investigate the effects and safety of fenofibrate on CVD and ESRD. The result after a 5-year follow-up showed fenofibrate reduced the total cardiovascular events and preserved renal function without any adverse effects.^135^ However, lowering the triglyceride levels might increase the serum creatine levels in the patients with eGFR <30 mL/min/1.73 m^2,^.^136^ Another agonist of PPARα, clofibric acid, acted to improve cardiac function and decrease the excretion of urine albumin but not serum creatine in 5/6 nephrectomy rats.^137,138^

PPARγ agonists, the thiazolidinedione (TZD) family, not only regulate lipid metabolism, but also protect the kidney from multiple damaging processes, such as inflammation, aging, and OS.^139^ A randomized controlled trial (RCT) results showed rosiglitazone inhibited the increase in albumin/creatinine ratio (ACR) as compared to metformin and preserved eGFR as compared to glyburide in type 2 diabetic patients.^140^ The prevention of CKD progression by TZDs has also been demonstrated in various animal studies.^141^ For example, pioglitazone preserved mitochondrial function and attenuated renal fibrosis in 5/6 nephrectomy rats and aging rats.^142,143^

#### CD36 antagonist

CD36 plays a key role in mediating the renal lipid uptake. Souza et al. revealed that apoA I-mimetic peptide 5A could attenuate renal fibrosis and reduce macrophage infiltration in the UUO mice. The peptide 5A treatment did not show fewer renal lesions than the CD36 knockout mice. This indicated that the peptide 5A might play a renal protective role by antagonizing CD36.^144^ The anti-CD36 antibody could inhibit the secretion of TGF-β1 induced by the advanced oxidation protein products (AOPPs) treatment.^145^ CD36 peptide (p93-110) that specifically inhibited the interaction of CD36 and TSP1 restored the FFA-induced apoptosis of podocytes.^102^

#### Mitochondrial protective drugs

Mitochondrion is a major place for FAO in TECs. Thus, protecting mitochondrion facilitates lipid metabolism keeping balance. A promising mitochondria-targeting drug, Szeto-Schiller peptide 31 (SS-31) specifically targeted cytochrome c and kept mitochondrial membrane potential abolished lipotoxicity, loss of podocytes and endothelial cells, glomerulosclerosis, and inflammation in HFD mice.^146^ Besides its role in HFD mice, the renal protective effects of SS-31 have also been demonstrated in various models, including warm ischemia-reperfusion, UUO, postischemic CKD, and aging mice.^147^ Mitochondria-targeting anti-oxidation drug, MitoQ, prevented the glomerular hypertrophy and mesangial expansion in db/db mice.^148^ A Chinese traditional medicine, berberine, could improve mitochondrial function and reduce lipid deposition in podocytes by promoting the PGC-1α expression.^149^ The PPAR and AMPK agonists discussed in other sections in this review could also promote mitochondrial biogenesis.^62^ The mitochondria-targeting drugs are well-reviewed elsewhere.^62,147,150^

#### PUFA

We have discussed the disadvantages of SFAs, however, not all FAs are bad for kidneys. PUFAs, especially, the long-chain n-3 PUFA, including DHA and eicosapentaenoic acid, are the good FAs. Various clinical trials and animal experiments have demonstrated that PUFAs could reduce albuminuria and ameliorate hypertension, inflammation, and glomerulosclerosis in DN.^151^ However, recent studies draw different conclusions. A meta-analysis revealed that the supplementation of n-3 PUFA caused a decrease in the cardiovascular death of CKD patients with hemodialysis and prevented ESRD among the CKD patients, who did not receive renal replacement.^152^ But, the evidence certainty of these conclusions was very low due to the small sample size, low event rates, and presence of moderate heterogeneity of the trials included in the analysis. An RCT study tested the effects of n-3 PUFA on renal injury in type 2 diabetic patients. The results suggested that, as compared to placebo, the n-3 PUFA treatment (4 g/day) had no effects on the excretion of urine albumin, serum markers of kidney function, and eGFR. Only urine neutrophil gelatinase-associated lipocalin levels decreased in the n-3 PUFA treated group.^153^ The results were consistent with the Vitamin D and Omega-3 Trial (VITAL), which concluded that the supplementation of vitamin D3 or omega-3 fatty acids could not preserve kidney function in type 2 diabetic patients.^154^ Although its monotherapy did not prevent CKD progression, the n-3 PUFA enhanced the effects of RAAS inhibitors in decreasing proteinuria and renal injury markers.^153^

#### Bile acid receptors agonist

The activation of bile acid receptors, including FXR and takeda G protein-coupled receptor 5 (TGR5) which increase FAO are promising therapeutic targets for CKD.^155^ FXR agonist GW4064 decreased glomerulosclerosis and interstitial fibrosis by inhibiting the SREBP1 expression.^156^ Selective FXR agonist INT-747 modulated lipid accumulation and improved renal injury, such as proteinuria, glomerulosclerosis, and tubulointerstitial fibrosis in streptozotocin (STZ)-induced DN mice.^70^ The possible mechanism might be inhibiting the transcriptional activity of NF-κB. A selective TGR5 agonist INT-777 induced mitochondrial biogenesis and then increased the FAO in obesity-associated nephropathy and DN.^157^ A dual FXR/TGR5 agonist INT-767 played a renal protective role in diabetes- and obesity-related kidney diseases by affecting multiple pathways related to the lipid metabolism, such as the upregulation of AMPK, SIRT1, PGC-1a, and Nrf-1.^71^

#### Other drugs targeting lipid metabolism

AMPK plays an important role in multiple lipid metabolism activities including enhancing FAO by regulating CPT1a expression and mitochondrial biogenesis, and inhibiting lipid synthesis. AMPK agonist A-769662 (A76) ameliorated renal fibrosis in 5/6 nephrectomy mice fed a high-protein (HP) diet.^59^ AICAR improved lipid accumulation, mitochondrial and lysosomal dysfunction, and renal fibrosis in HFD mice.^158^ Anthocyanins and resveratrol decreased albuminuria and improved the expansion of the glomerular matrix and inflammation in db/db mice via the phosphorylation of AMPK.^159,160^ A recent study demonstrated that a small molecule TW-37 could inhibit the uptake of KIM-1-mediated PA-albumin and ameliorate renal inflammation and fibrosis in vivo.^76^ ABCA1 inducer A30 ameliorates podocyte injury and CKD by reducing the cardiolipin oxidation.^79^

#### Nonpharmacological therapy

Besides artificial drug therapy, lifestyle modification is also important for preventing CKD progression. A meta-analysis summarized 17 studies that observed the effects of body weight loss through lifestyle, pharmacological or surgical intervention on the CKD patients, who were overweight or obese. The results showed a decrease in the level of LDL-cholesterol due to weight loss. However, the total cholesterol, HDL-cholesterol, and C-reactive protein had no significant changes. The effects of weight loss on the reduction of proteinuria, blood pressure, and cardiovascular death are uncertain.^161^ An RCT study investigated the additive anti-proteinuria effects of weight loss on angiotensin II receptor blockers (ARBs) in hypertensive CKD patients.^162^ Authors revealed that weight loss was beneficial for proteinuria reducing the effect of ARBs. Another trial also observed a significant reduction of serum creatinine and cystatin C levels in a short-term intensive weight reduction intervention group of DN patients.^163^

## Signaling pathways and targeted therapies in oxidative stress

**Fig. 4:**
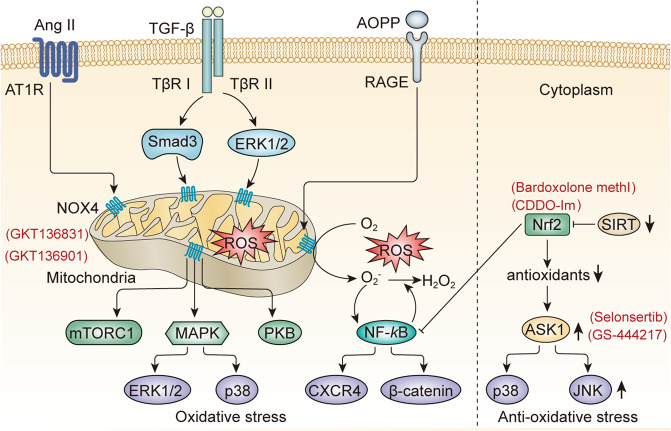
Key signaling pathways mediated oxidative stress. NOXs are core enzymes mediated oxidants production. Nox4 is upregulated in the kidney by Ang II and TGF-β pathways or by oxidation end products AOPP. Upregulated Nox4 induces excessive ROS accumulation and activates downstream pathways including ERK, mTORC, PKB, and MAPK signaling. Excessive ROS activates the NF-κB pathway, inducing chronic inflammation and renal fibrosis by upregulating the expression of CXCR4 and β-catenin. As positive feedback, NF-κB activation increased the expression of NOX and NOS to create more ROS. Downregulation of the SIRT family inhibits Nrf2, which is a positive regulator of antioxidant proteins and an inhibitor of NF-κB. The reduction of antioxidants increases the expression of ASK1 that activates the p38 and JNK. Red fonts in brackets are inhibitors. NOX nicotinamide adenine dinucleotide phosphate (NADPH) oxidase, NOS nitric oxide synthase, Ang II angiotensin II, TGF-β transforming growth factor-β, ROS reactive oxygen species, AOPP advanced oxidation protein products RAGE receptor of advanced glycation end products, ERK extracellular regulated protein kinases, mTORC mammalian target of rapamycin complex, PKB protein kinase B, MAPK mitogen-activated protein kinase, NF-κB nuclear factor kappa-B, SIRT sirtuins, Nrf2 nuclear factor erythroid 2-related factor 2

Reactive oxygen is a side product of oxidative phosphorylation in the mitochondrion. And it can be cleared by antioxidants such as superoxide dismutase (SOD), and glutathione. However, when the balance between oxidants and antioxidants is disturbed, excessive oxidants including ROS and reactive nitrogen species (RNS) lead to renal injury. And the process is regarded as the OS of the kidney. As high oxygen consume organ, the kidney is vulnerable to OS damage. In this part, we will discuss the signaling pathways of oxidants generation, OS, and the targeted therapies for CKD (Fig. 4).

### Signaling pathways of oxidative stress in CKD

ROS, which includes the superoxide anion (O2^–^), hydrogen peroxide (H_2_O_2_), and hydroxyl radicals (OH), are mainly catalyzed by nicotinamide adenine dinucleotide phosphate (NADPH) oxidase (NOX) and xanthine oxidase (XO) in mitochondria.^164^ Among NOX family members, Nox4 has been best characterized in the kidney. Nox4 was proved to be upregulated in most renal resident cells including TECs, podocytes, mesangial cells, and endothelial cells after renal injury, and promoted the progression of kidney disease.^165^ Smad3 and extracellular-signal-regulated kinase (ERK1/2) signaling activation triggered by TGF-β increased the expression of Nox4.^166,167^ Ang II treatment also induced the upregulation of Nox4, and the successive ROS accumulation activated ERK, Akt, PKB, and eGFR signaling.^165,168,169^ Podocyte-specific induction of Nox4 in vivo stimulated ER stress, and expression of hypoxia-inducible factor-1α (HIF-1α) and TGF-β, leading to glomerulosclerosis and albuminuria.^170,171^ And podocyte-specific Nox4 deletion attenuated albuminuria, glomerulosclerosis, mesangial expansion, as well as glomerular basement membrane thickness by inhibiting renal ROS, glomerular MCP-1, and protein kinase C alpha (PKC-α).^172^ The upregulated Nox4 and H_2_O_2_ activated mTORC1, which contributed to salt-induced hypertension and renal injury in the SS rat model.^173^ Excessive ROS triggered inflammatory NF-κB signaling and promoted the progression of renal fibrosis.^174,175^ Circularly, NF-κB activation increased the expression of NOX and nitric oxide synthases (NOS) which were core enzymes that mediated oxidants production.^175^ However, Nox4 knockout mice enhanced renal fibrosis and OS after UUO injury.^176^ It indicated too much or too less ROS can be harmful to the kidney. Other Nox members were also studied in the kidney. Nox2 and Nox5 were upregulated in Ang II treated podocytes.^165^ Consistently, Ang II receptor blockers inhibited Nox2 expression and were accompanied by increased SOD expression, decreased OS, and reduced renal and cardiac fibrosis.^177^ Specific overexpression of human Nox5 in vascular smooth muscle cells or mesangial cells led to glomerulosclerosis, inflammation, and renal fibrosis in diabetic mice.^178^

NOS is necessary for the synthesis of RNS that principally includes NO, peroxynitrite (ONOO−), nitrotyrosine, and nitrosothiols. NOS has three isoforms. Neuronal NOS (nNOS) and endothelial NOS (eNOS) are constitutively expressed by central and peripheral neurons and endothelial cells, respectively. However, inducible NOS (iNOS) is evoked by inflammation.^179^ In a healthy kidney, nNOS is expressed in cortical tubules but not in glomeruli, whereas eNOS is expressed in glomeruli but not in tubules.^180^ Increased iNOS in injured kidneys was associated with inflammation in CKD animal models.^181^ Dual CB1 receptor/iNOS antagonist, MRI-1867 decreased renal inflammation, fibrosis, and OS of obesity-related kidney disease.^182^ However, the studies about NOS in CKD are not well understood currently.

Except for the direct damage to the kidney, oxidants modified proteins, lipids, and DNA to form oxidation end products. AOPP bound to the receptor of advanced glycation end products and triggered the activation of Nox2, leading to the production of ROS. The accumulated ROS activated p65 NF-κB, increasing the generation of Wnt ligands and subsequent cytoplasmic β-catenin accumulation. The nuclear translocation of β-catenin due to excessive cytoplasmic storage triggered podocyte injury and albumin urine.^183^ The podocyte injury caused by AOPP treatment was also mediated by C-X-C chemokine receptor type 4 via p65 NF-κB signaling.^184^ Lipid peroxidation products cause DNA damage by synthesizing readily diffusible biofilm species such as aldehydes such as malondialdehyde or 4-hydroxynonenal. Membrane lipid peroxidation disrupted cellular and mitochondrial membrane barriers, interfering with electron transport, which in turn reduced ATP production.^185^

OS is not only due to the accumulation of ROS, but also the decrease of antioxidants. In OS, without the repression of antioxidant protein thioredoxin, apoptosis signal-regulating kinase 1 (ASK1) was phosphorylated and activated the downstream MAPKs p38 and JNK, leading to progressive kidney injury.^186^ Nuclear factor erythroid 2-related factor 2 (Nrf2) is a central redox-sensitive transcription factor that positively regulates antioxidant proteins including glutathione S-transferase, heme oxygenase-1, catalase, γ-glutamylcysteine synthetase, and NAD(P)H quinone oxidoreductase.^187^ Kelch-like ECH-associated protein-1 (Keap1) is a core repressor of Nrf2. Keap1 binds to Nrf2 and then leads to the polyubiquitination and subsequent proteasomal degradation of Nrf2. The renal protective role of Nrf2 was proved in multiple CKD animal models.^188^ Nrf2 deletion aggravated renal fibrosis induced by UUO, OS, and glomerulosclerosis of STZ-induced DN, renal damage of lupus nephritis.^189–191^ Besides regulating antioxidants, inhibiting inflammation is another mechanism of Nrf2 for protecting renal from injury. Nrf2 blocked the activation of the master driver of the inflammatory response, the NF-κB signaling pathway, via multiple different mechanisms. For example, Keap1 decreased the activation of upstream kinase IKKβ. And Nrf2 competed with NF-κB for binding to transcriptional coactivator CBP, resulting in the reduction of downstream genes of NF-κB.^187^

Sirtuins (SIRT) family is crucial to negatively regulate OS. Podocyte-specific SIRT1 overexpression attenuated podocyte loss and reduced OS in diabetes-induced nephropathy.^192^ Increased cyclooxygenase-2 (COX2) and its derivative PGE2 regulated by SIRT1 protected mouse renal medullary interstitial cells from OS.^193^ The natural antioxidant, resveratrol (RSV), improved manganese-superoxide dismutase (Mn-SOD) dysfunction in AMPK/SIRT1 dependent way and prevented the progression of diabetic kidney disease.^194^ RSV also prevented the aging pathologic changes in kidneys by elevating the level of Nrf2 and SIRT1.^195^ SIRT3 protected TECs from apoptosis by inhibiting ROS accumulation and ROS-sensitive Akt/FoxO signaling pathway.^196^ Overexpression of SIRT6 significantly inhibited Ang II-induced vascular endothelial cells apoptosis and ROS generation by upregulating the expression of the Nrf2 and its antioxidative downstreams.^197^ And SIRT6 played a protective role in OS by other pathways, such as decreasing the phosphorylation of AKT, increasing the phosphorylation of AMPK, and the levels of FoxO3α.^198^

### The antioxidative stress therapies

Supplementing antioxidants is no doubt a direct approach to ameliorating OS. Pyridoxamine dihydrochloride (Pyridorin) that is a derivative of vitamin B6 is supposed to be a candidate for CKD treatments by scavenging ROS and inhibiting the formation of advanced glycation end products.^199^ However, a double-blind, randomized, placebo-controlled trial of Pyridorin failed to show the serum creatinine decreasing effect compared to the placebo group in type 2 DN patients with proteinuria.^200^ A phase 3 clinical trial (the PIONEER trial, NCT02156843) was conducted to evaluate the safety and efficacy of oral Pyridorin in reducing the risk of progression of DN. And the results are not reported yet.

We have discussed the important role of Nox, especially Nox4 in the OS and progression of kidney disease. The inhibitors of the Nox family have been tested in clinical trials and animals to evaluate the renoprotective effect. GKT136901, a Nox1/4 inhibitor treatment reduced albuminuria and the development of nephropathy in db/db (diabetic) mice by decreasing renal ERK1/2 phosphorylation and oxidative damage.^201^ Another Nox1/4 inhibitor, GKT137831 also exhibited the renal beneficial effect.^171,202^ Since GKT137831 achieved satisfactory renal protection in various CKD animal models, a multicenter, randomized, placebo-controlled trial about GKT137831 was conducted in type 1 diabetes-related kidney disease patients. The primary outcome of this trial was comparing the urine albumin to creatinine ratio and eGFR in different groups.^203^ The outcome of this not ended trial will provide strong evidence for the efficacy of Nox inhibitors in CKD.

Bardoxolone methyl is an inducer of Nrf2 which acts to release Nrf2 from the depression of Keap1. Elegant clinical trials were conducted to evaluate the effect of Bardoxolone methyl on CKD patients. The BEAM study (NCT00811889) showed that bardoxolone methyl improved the eGFR of CKD patients.^204^ However, proteinuria and blood pressure were elevated. A phase 3 clinical trial, the BEACON study (NCT01351675), confirmed the results and revealed that the risk of ESRD or death from cardiovascular causes was not reduced even higher in the bardoxolone methyl-treated group than in the placebo group.^205^ The other phase 3 clinical trials about bardoxolone methyl are undergoing in patients with autosomal dominant polycystic kidney disease (ADPKD) (NCT03918447) and Alport Syndrome (NCT03019185). The renal unfriendly effect of Nrf2were also repeated in animal studies. Tan et al. showed that Keap1 deficiency in vivo elevated Nrf2 signaling and aggravated proteinuria evoked by adriamycin, Ang II, or protein overload. And Nrf2 inducer CDDO-Im significantly increased the proteinuria of Ang II treated mice. Conversely, Nrf2 knockout mice ameliorated proteinuria induced by Ang II.^206^

GS-444217 was designed to inhibit ASK1 and was proved to halt the decline of renal function and renal fibrosis. Furthermore, GS-444217 strengthened the anti-glomerulosclerosis and albuminuria effects of the angiotensin-converting enzyme inhibitor (ACEi).^186^ Another ASK1 inhibitor, selonsertib was evaluated in moderate-to-advanced diabetic kidney disease.^207^ Randomized, dose-ranging, placebo-controlled phase 2 trial results showed selonsertib had no dose-dependent adverse effects over 48 weeks. And the rate of eGFR decline was reduced compared to placebo between 4 and 48 weeks after excluding confounding factors. However, acute reductions in creatinine secretion at weeks 0–4 in the high-dose group prevented the trial from meeting the primary endpoint. Urine ACRs also did not differ between the selonsertib and placebo groups. From the above clinical trial results of drugs for OS, it can be concluded that signaling pathways of OS are potential targets of CKD, but new targets and treatments remain to be discovered.

## Signaling pathways of chronic inflammation

**Fig. 5:**
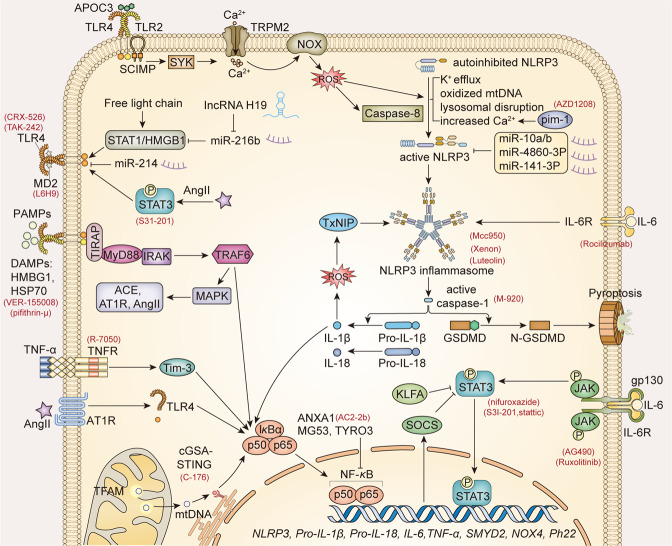
Signaling pathways regulated inflammation in CKD. NF-κB is a central transcriptional factor of inflammation. TNF-α binds to the TNF receptor and activates NF-κB in Tim-3 dependent way. TLR4 involves in the activation of NF-κB induced by Ang II. mtDNA released from mitochondria caused by TFAM deletion triggers cGAS-STING DNA-sensing signaling activation and the STING-dependent NF-κB activation. DAMPs like HMGB1 and HSPs bind to TLR2 or TLR4 and attract dimerization. Activated TLR4 recruits adaptor proteins including TIRAP, MyD88, TRAM, IRAK, TRIF to activate NF-κB. TLR signaling regulates the production of NLRP3, pro-IL-1β, and pro-IL-18 that is the priming process of NLRP3 inflammasome formation in NF-κB-dependent way. NLRP3 inflammasome is assembled by NLRP3, the adaptor protein ASC and active caspase 1. ROS, K+ efflux, oxidized mitochondrial DNA release, and ion fluxes activate NLRP3 inflammasome and the successive caspase 1. Activated caspase 1 cut pro-IL-1 and pro-IL-18 to produce mature IL-1 and IL-18. It also cleaves and activates the cytoplasmic gasdermin proteins, and the activated gasdermin proteins are translocated to the membrane to constitute holes for IL-1 and IL-18 release. ApoC3 induced the activation of NRLP3 inflammasome in caspase-8-dependent way and the subsequent IL-1β release. IL-6 binds to the membrane IL-6 receptor (IL-6R), and recruits and homodimerizes signal transducer glycoprotein 130 (gp130). gp130 mediates the activation of the JAK and leads to the phosphorylation of STAT. SOCS can be upregulated by JAK-STAT signaling activation and acts as the negative feedback of JAK-STAT signaling. Red fonts in brackets are inhibitors. NF-κB nuclear factor kappa-B, JAK Janus kinase, STAT signal transducer and activator of transcription, NRLP3 NOD-like receptor protein 3, TLR toll-like receptor, TNF-α tumor necrosis factor-α, Ang II angiotensin II, IL interleukin, DAMPs danger-associated molecular patterns, ROS reactive oxygen species, TFAM mitochondrial transcription factor A, ASC apoptosis-associated speck-like protein containing a CARD, SOCS suppressor of cytokine signaling, TIRAP TIR domain-containing adaptor protein, MyD88 myeloid differentiation factor 88, TRAM TRIF-related adaptor molecule, IRAK interleukin receptor-associated kinase, TRIF TIR domain-containing adaptor inducing interferon, HSPs heat shock proteins, HMGB1 high-mobility group box 1

Inflammation plays a critical role in the initiation and progression of renal fibrosis. Proinflammatory and profibrotic cytokines and inflammatory cells which include immune cells from bone marrow and locally damaged resident renal cells constitute the inflammatory microenvironment. Macrophages are the best-characterized immune cells in renal inflammation.^208^ M1 and M2 are two extreme phenotypes of macrophages. M1 proinflammatory macrophages produce ROS, TNF-α, and IL-1β to amplify inflammation and mediate acute kidney inflammation. While the repairing phase, the majority of macrophages convert into reparative M2 phenotype that acts to resist acute inflammation by secreting anti-inflammatory cytokines like IL-10 and insulin-like growth factor-1.^209^ However, the persistent existence of M2 macrophages triggers immoderate repair which is characterized by excessive ECM deposition. And M1 macrophages also participate in chronic inflammation and renal fibrosis.^12,210,211^ Mechanistically, macrophages produce a large number of cytokines and growth factors like TGF-β1 to activate myofibroblasts from multiple sources such as fibroblasts, TECs, and pericytes.^212^ Macrophages contribute to the pool of myofibroblasts directly through macrophage to mesenchymal transition (MMT).^213,214^ And macrophages damage vascular endothelial cells and enhance hypoxia injury.^12^ The role of T cells in renal disease has been revealed extensively.^215^ For example, CD28^−^ T cells accumulate in patients with CKD and anti-immune disease. Senescent CD28^−^ T cells secret lots of inflammatory cytokines such as IFNγ and TNF-α, and express cytotoxic effector molecules such as granzyme B and perforin to promote tissue inflammation and injury.^216^ Helper CD4^+^ T lymphocytes (Th) deletion reduced renal fibrosis and reconstitution. CD4^+^ T cells, especially Th2 cells contributed to the progression of renal fibrosis in UUO mice by secreting profibrotic Th2 cytokines such as IL-4 and IL-13.^217,218^ Cytotoxic CD8^+^ T lymphocytes enter into glomeruli and kill podocytes only when Bowman’s capsule is disrupted.^219^ However, CD8^+^ T cells play a renal protective role in ADPKD.^220^ Fibrocytes are the important collagen production immune cells.^221^ The mechanisms of fibrocytes promoting renal inflammation and fibrosis are producing inflammatory cytokines such as TNF-α and MCP-1 and profibrotic cytokines like TGF-β, and converting into myofibroblasts directly.^12^ NF-κB pathway, JAK-STAT signaling, inflammasome, TLR signaling, and other signalings such as cGAS-STING signaling participate in the activation of inflammatory cells and the generation of inflammatory cytokines (Fig. 5). In this part, we will discuss major signaling pathways that mediated the activation of immune cells and the promising therapies for CKD based on inflammation.

### Major signaling pathways participate in the activation of immune cells

#### NF-κB pathway

As transcription factors, the NF-κB family regulates the induction and resolution of numerous inflammatory genes which are crucial to kidney disease by binding to the κB elements in promotor and enhancer. RelA (also called p65), RelB, c-Rel, p50 (also called NF-κB1), and p52 (also called NF-κB2) are five members of the NF-κB family.^222^ The shared Rel homology domain is the binding domain for Rel proteins to form the heterodimers of NF-κB. RelA, RelB, and c-Rel contain a DNA-binding domain that is necessary for the transcription of target genes. p50 and p52 that lack DNA-binding domain form heterodimers with other Rel proteins, and modulate the DNA-binding activity of NF-κB.^223^ The p65/p50 dimer is the most studied heterodimer in kidney disease. In healthy kidneys, NF-κB is anchored in the cytoplasm with the assistance of Inhibitors of κB (IκB) proteins including IκBα, IκBβ, p100, p105, and IκBε. The inhibitor of the κB kinase (IKK) complex is composed of two catalytic subunits, IKKα (IKK1) and IKKβ(IKK2), and a regulatory subunit, IKKγ. When stimulus triggers the cascade of NF-κB signaling, IKKs are phosphorylated by TGFβ-activated kinase 1 (TAK1), and then promote the ubiquitination and degradation of IκB.^224^ Then, NF-κB is released from IκB and transfers into the nucleus to regulate the transcription of target genes. The canonical NF-κB pathway is mainly mediated by IκBα. In the activation of the noncanonical NF-κB pathway, NF-κB inducing kinase (NIK) and IKKα phosphorylate p100 to release RelB.^223^ NF-κB signaling has been widely investigated in CKD. It is concluded that NF-κB signaling is a key mediator of renal inflammation both in CKD animals and CKD patients. The role of NF-κB signaling in CKD has been reviewed elsewhere.^223–225^ In this part, we update the regulation and crosstalk of NF-κB signaling in CKD with the latest research.

TNF-α and angiotensin II are the strong stimuli of NF-κB signaling. TNF-α binds to the TNF receptor and activates NF-κB. The increased transcription of TNF-α and SMYD2 can be triggered by NF-κB. SMYD2 promoted methylation and phosphorylation of p65 at K221 and K218, leading to the progression of ADPKD.^226^ The positive feedback loops between SMYD2 and NF-κB enhanced renal inflammation. Tim-3 mediated the activation of NF-κB induced by TNF-α.^227^ TNF receptor-1 inhibitor, R-7050 reduced renal damage and fibrosis in wild-type mice and mice with Krüppel-like factor 4 (KLF4)-deficient macrophages.^210^ Another TNF superfamily (TNFSF) member, TWEAK was proved to worsen the phenotype of ADPKD mice and renal fibrosis of UUO mice by activating NF-κB signaling.^228,229^ TWEAK is also an activator of nonclassical NF-κB signaling, and is the only cytokine demonstrated to activate nonclassical NF-κB signaling in tubules currently. NIK deficiency disturbed TWEAK-induced CCL19, CCL21, and CXCL10 generation in TECs.^230^ As a strong inflammatory cytokine, TNF-α plays a prominent role in renal inflammation and fibrosis by activating other multiple pathways, not only the NF-κB pathway. For example, TNF-α induced the expression of retinoic acid receptor responder protein 1 (RARRES1). And the soluble RARRES1 activated p53 and induced podocytes apoptosis by binding with and inhibiting RIO kinase 1 (RIOK1).^231^

Angiotensin II binds to angiotensin II type 1 receptor (AT1R) and activates AT1R, which promotes phosphorylation of p65 at Ser536, and then triggers the transcription of inflammatory factors like MCP-1, IL-8, IL-6, TNFα, and IL-17A.^232^ However, AT1R deficiency did not reverse inflammation completely due to the compensation of AT2R. Only both AT1R and AT2R antagonist applications blocked renal monocyte infiltration and NF-κB activation of UUO mice.^233^ The other signaling pathways are involved in the regulation of NF-κB. The receptor activator of NF-κB (RANK) upregulation induced by high glucose activated NF-κB and then increased the transcription of inflammatory cytokines, and OS-associated genes, Nox4 and its obligate partner, P22phox, resulting in the podocyte injury in DN.^234^ NF-κB is negatively regulated by some proteins. Cytoplastic annexin A1 (ANXA1) bound to NF-κB subunit p65 and inhibited its nucleus translocation. Thus, ANXA1 overexpression ameliorated kidney injuries, albuminuria, glomerulosclerosis, tubulointerstitial fibrosis, and kidney inflammation by inhibiting NF-κB signaling.^235^ The consistent results were obtained from ANXA1 mimetic peptide, Ac2-26 treated diabetic kidney disease mice. MG53 (also named TRIM72) was also proved to inhibit the activation of NF-κB and attenuated renal fibrosis by binding to p65 and blocking its nucleus translocation.^236^ The activation of NF-κB decreased the level of TYRO3 which was a podocyte protective factor in glomerular disease.^237^ Protein S inhibited the TNF-α induced NF-κB activation in a TYRO3 receptor-dependent way.^238^

Epigenetic regulation like microRNAs (miRNAs) that are a class of small noncoding RNAs that regulate gene expression through binding target mRNAs plays an important role in NF-κB activation. Angiotensin II treatment increased circulating miR-103a-3p expression, resulting in the reduction of serine/threonine-protein kinase of glomerular endothelial cells that exerted anti-inflammatory effects by inhibiting NF-κB/p65 activation.^239^ Methyltransferase-like 3 promoted m6A modification of miR-21-5p. The maturation of miR-21-5p activated NF-κB via SPRX1/ERK signaling and enhanced renal fibrosis and inflammation of UUO mice.^240^ TECs derived miR-19b-3p abundant exosome potentiated NF-κB signaling of macrophages by abolishing the repression of suppressor of cytokine signaling-1 (SOCS1).^241,242^ miR-802 inhibited the expression of NF-κB repressing factor and promoted the renal injury of obesity-associated nephropathy by activating NF-κB.^243^ miR-373 induced by TGF-β targeted to SIRT1 which inhibited NF-κB and enhanced renal fibrosis.^244^ miR-146a inhibited classical and nonclassical NF-κB signaling pathways and ameliorated lupus nephropathy and DN.^245,246^

#### JAK-STAT signaling

The Janus kinase (JAK)-signal transducer of activators of transcription (STAT) pathways was discovered in the anti-viral process of interferons. The phosphorylation of JAK activates STAT directly and then the transcription factor STAT regulates targeted genes in the nucleus.^247^ This two-step pathway delivers transmembrane signals to the nucleus rapidly. JAK family has four members including JAK1, JAK2, JAK3, and receptor tyrosine kinase 2 (TYK2). And there are seven STATs such as STAT1 to 4, 5a, 5b, and 6. More than 50 cytokines and growth factors are regulated by JAK-STAT signaling. All members of JAK-STAT signaling have been found in the damaged kidney.^248,249^ In human kidney biopsy of high inflammatory kidney diseases such as lupus nephritis, DN, IgA nephropathy, and FSGS, JAK, and STAT, and the inflammatory factors including IL4R, IL6R, IL-13, and interferon-gamma that involved in JAK-STAT signaling were upregulated.^250–252^ Peripheral blood monocytes (PBMCs) from patients with IgA nephropathy have upregulated STAT production after cytokines stimulation.^251^ JAK-STAT upregulation in podocytes, TECs, and mesangial cells was associated with the progression of DN.^253^ Podocyte-specific JAK2 overexpression aggravated albuminuria, mesangial expansion, glomerular basement membrane thickening, and tubulointerstitial fibrosis of Akita diabetic mice. Oral JAK1/2 inhibitor, LN3103801 treatment alleviated the pathology changes of JAK2 overexpression in diabetic mice.^254^ The overexpression of E3 ubiquitin ligase casitas B-lineage lymphoma (Cbl) degraded JAK2, and inactivated the successive STAT4 and the targeted Runx3 transcription, resulting in the inhibition of endothelial dysfunction by increasing NO production.^255^ STAT6 was increased in TECs and STAT6 deficiency attenuated UUO-induced renal fibrosis. A natural carotenoid extracted from the seeds of *Bixa Orellana*, bixin, ameliorated kidney interstitial fibrosis by suppressing the phosphorylation and stability of STAT6.^256^ JAK3-STAT6 signaling pathway mediated monocytes from bone marrow to myofibroblasts transition. Wild-type mice engrafted with STAT6(-/-) bone marrow cells displayed fewer myofibroblasts infiltration and ECM deposition in the obstructed kidneys.^257^ And UUO mice treated with the inhibitor of JAK3, CP690,550 showed similar results. However, the JAK/STAT inhibitor, tofacitinib, did not attenuate uric acid crystal-induced inflammation and CKD progression.^250^

IL-6 is a key proinflammatory factor in innate and adaptive immune responses. IL-6 binds to the membrane IL-6 receptor (IL-6R), and recruits and homodimerizes signal transducer glycoprotein 130 (gp130). Membrane proximal domains (Box domains 1 and 2) of gp130 mediate the activation of the JAK and leads to the phosphorylation of STAT.^258^ Recombinant gp130 linked with Fc competitively binds to IL-6/IL-6R complex with endogenous gp130 and abolishes the transduction of IL-6 signaling. Fc-gp130 administration prevented renal fibrosis by inhibiting the activation of STAT3.^259^ Post-transcriptional modification such as acetylation of STAT enhanced the dimerization and nucleus translocation of STAT. Inhibited STAT3 acetylation reduced albuminuria and kidney injury of diabetic db/db mouse.^260^ The SOCS is a prominent negative feedback protein of JAK-STAT signaling. SOCS can be upregulated by JAK-STAT signaling activation. The kinase inhibitory region of SOCS1 binds to the substrate-binding groove of JAK with high specificity and acts as a pseudo substrate for JAK kinases.^261^ Overexpressing SOCS1 or SOCS3 with adenovirus mitigated interstitial fibrosis, macrophage infiltration, and proteinuria of DN by suppressing STAT1 and STAT3 activation.^262^ KLF4 that was a DNA-binding transcription regulator suppressed STAT3 transcriptional activity by binding to pSTAT3 (Tyr705). Podocyte-specific knockout KLF4 activated STAT signaling, resulting in podocytes injury, and parietal epithelial cells activation.^263,264^

#### The activation of the NLRP3 inflammasome

The inflammasome is a multi-protein complex assembled by cytoplasmic pattern recognition receptors (PRRs) that sense pathogen-associated molecular patterns (PAMPs) or danger-associated molecular patterns (DAMPs) and is an important part of the innate immune system. NOD-like receptors (NLRs), TLRs, Rig-I-like receptors, absent in melanoma 2 (AIM2)-like receptors (ALRs), and C-type lectin receptors are the five families of PRR.^265^ Only proteins from the NLR and ALR families form inflammasomes. The expression of inflammasome components in the kidney was reviewed elsewhere.^266^ NLRP3 inflammasome is the best-characterized inflammasome in CKD.^267^ NLRP3 inflammasome is assembled by NLRP3, the adaptor protein apoptosis-associated speck-like protein containing a CARD (ASC) and active caspase 1. ROS, K^+^ efflux, oxidized mitochondrial DNA release, and ion fluxes due to lysosomal disruption activate NLRP3 inflammasome and the successive caspase 1 which executes inflammatory factors production and cell death.^268^ Activated caspase 1 cut pro-IL-1 and pro-IL-18 to produce mature IL-1 and IL-18. It also cleaves and activates the cytoplasmic gasdermin proteins, and the activated gasdermin proteins are translocated to the membrane to constitute holes, leading to cell swelling, cytoplasmic outflow, cell membrane rupture, and pyroptosis finally.^269^ The inflammatory factors such as IL-1, and IL-18 outflow from the holes composed of gasdermin.^270^

The proinflammatory and profibrotic effect of NLRP3 inflammasome has been confirmed in multiple animal models of kidney diseases such as UUO, angiotensin II-induced hypertensive kidney injury, DN, glomerulonephritis, and 5/6 nephrectomy.^266^ Then we summarize the regulation of NLRP3 inflammasome and the following pyroptosis in CKD. IL-36 cytokines facilitated NLRP3 inflammasome activation in an IL-36 receptor-dependent way and contributed to renal inflammation and fibrosis of UUO mice.^271^ Apolipoprotein C3 (ApoC3) activated human monocytes and promoted renal injury from unilateral ureter obstruction in humanized mice.^272^ Mechanically, ApoC3 activated Syk through TLR2 or TLR4 in the assistant of transmembrane protein SCIMP which contains immunoreceptor tyrosine-based activation motifs. Syk-mediated calcium influx through TRPM2 evoked NAPDH oxidase that catalyzed the generation of ROS. Finally, ApoC3 induced the activation of NRLP3 inflammasome in caspase-8 dependent way and the subsequent IL-1β release. ApoC3 also induced the cascade of NF-κB signaling and MAPK signaling. The pyroptosis can be triggered not only by caspase 1 but also by caspase-3. Caspase-3 and GSDME were elevated in TECs and promoted the progression of obstructive nephropathy by enhancing TECs’ pyroptosis. And this process could be induced by TNF-α and caused the release of high-mobility group box 1 (HMGB1) that was a nuclear protein acting as a DAMP.^273^ The free HMGB1 recruits macrophages and neutrophils from bone marrow and triggers NLRP3 inflammasome activation of these immune cells.^274^ The upregulated NLRP3 inflammasome/IL-1β cascade induced NF-κB p65 activation, mitochondrial ROS generation, and lipid accumulation of podocytes.^275^ Thioredoxin (TRX)-interacting protein (TXNIP) dissociates from TRX in a ROS-sensitive manner, and binds to and activates NLRP3 inflammasome.^276^ Mitochondria ROS (mtROS) generated in TECs under the treatment of high glucose decreased TRX expression and released TXNIP to activate NLRP3 inflammasome, resulting in tubular damage in the kidneys of patients with DN.^277^ And the NLRP3 activation and mtROS generation were regulated by increased CD36 expression in TECs.^278^ Pim-1 elevated in renal lysates from mice with lupus nephritis, PBMCs from patients with SLE, and renal biopsy tissue from patients with lupus nephritis. The upregulated Pim-1 increased the intracellular Ca^2+^ of podocytes which modulated NLRP3 inflammasome activation. And Pim-1 inhibitor AZD1208 ameliorated proteinuria, glomerulonephritis, renal immune complex deposits, and serum anti-dsDNA antibody levels in mice with lupus nephritis.^279^ Post transcription modification like miR-10a/b inhibited the formation of the NLRP3 inflammasome by binding to the 3’-untranslated region of NLRP3 mRNA.^280^ mir-486a-3p and miR-141-3p showed similar suppression effects of NLRP3 and protected kidneys from chronic injury.^281,282^

#### Toll-like receptor signaling

TLRs are one of the members of PRRs. Human TLRs have 10 isoforms including TLR1-10. Different from NLR that we discussed above, TLRs including TLR1, 2, 4, 5, and 6 are transmembrane proteins with various leucine-rich repeats which are the recognition domain of PAMPs and DAMPs. TLRs are predominantly expressed by immune cells like macrophages, T cells, B cells, NK cells, and dendritic cells.^283^ Resident renal cells such as TECs, podocytes, mesangial cells, and endothelial cells are also expressed TLRs when suffering from striking.^284^ DAMPs like HMGB1, and heat shock proteins (HSPs) bind to TLR2 or TLR4 and attract dimerization. Activated TLR4 recruits adaptor proteins including TIR domain-containing adaptor protein, myeloid differentiation factor 88, TIR domain-containing adaptor inducing interferon (TRIF)-related adaptor molecule, interleukin receptor-associated kinase, TRIF to mediate the production of ROS and the activation of NF-κB.^253,285^ TLR signaling induces NF-κB activation and regulates the production of NLRP3, pro-IL-1β, and pro-IL-18 which is the priming process of NLRP3 inflammasome formation.

TLRs play an important role in renal inflammation and progressive renal fibrosis. The research on TLRs in kidney diseases has been reviewed elsewhere.^286,287^ In summary, TLRs (mainly TLR4 and TLR2) activation promotes resident renal cells injury, immune cells derived from bone marrow recruitment, and the expression of inflammatory factors such as MCP-1, IL-6, IL-8, IL-1β in NF-κB/NLRP3 inflammasome signaling and MAPK signaling-dependent way. Here, we update the effect and regulation of TLRs in CKD. TLR4 deficiency in vivo or in vitro dampened NF-κB activation caused by angiotensin II treatment, thus decreasing renal inflammation and OS.^288^ AT1R blocker losartan reduced the expression of TLR4.^289^ STAT3 also mediated the activation of TLR4 induced by angiotensin II in renal proximal tubular cells, and STAT3 inhibitor S31-201 normalized renal fibrosis and dysfunction of hypertensive kidney disease.^290^ Free light chains such as κ and λ chains induced proximal tubule cell injury by promoting HMGB1 release and STAT1 upregulation, causing the expression of TLR2, TLR4, TLR6, and downstream proinflammatory cytokines.^291^ The proinflammatory TLR4/IFN-γ/STAT1 pathways transactivated the miR-214 gene which inhibited TLR4 directly in turn.^292^ The upregulated long noncoding RNA H19 in the injured renal tubular cells caused by calcium oxalate (CaOx) crystals countered miR-216b which inhibited HMGB1 expression by directly binding its 3’-untranslated region.^293^ Thus, an RNA H19 induced the expression of HMGB1, and the following TLR4 and NF-κB. The role of the co-receptor of TLR4 in innate immunity, MD2 had been revealed in DN. MD2 deficiency mice or MD2 inhibitor L6H9 abolished the activation of MAPK signaling and the downstream ACE, angiotensin receptors, and angiotensin II expression in proximal tubular cells.^294^ The co-receptors of TLR4 are diverse and mediate different even opposite outcomes. Biglycan evoked the cooperation of CD44 and TLR4, increased M1 macrophages autophagy and renal M2 macrophages infiltration, and reduced tubular damage after ischemia/reperfusion injury.^295^ PD is one of the renal replaced therapies for ESRD patients; however, peritoneal membrane fibrosis causes dialysis failure. TLR2 and TLR4 mediated profibrotic and proinflammatory responses of peritoneal membrane fibrosis. And the harmful effect of TLR2/4 was blocked by the TLRs inhibitor, soluble TLR2.^296,297^

#### Other signaling pathways

The cyclic GMP-AMP synthase (cGAS) – stimulator of interferon genes (STING) signaling plays a key role in innate immune defense. DNA sensor, cGAS is motivated by pathogenic or endogenous DNA and activates STING and the downstream type I interferon (IFN) gene transcription.^298^ mtDNA released from mitochondria caused by TFAM deletion triggered cGAS-STING DNA-sensing signaling activation and the STING-dependent NF-κB activation. TECs specific knockout STING or STING specific inhibitor C-176 treatment attenuated inflammation and the functional and structural changes of renal fibrosis.^299^ mtDNA release can be triggered by the loss of disulfide-bond A oxidoreductase-like protein (DsbA-L) as well. DsbA-L deficiency promoted inflammation and insulin resistance by activating the cGAS-STING pathway in adipose tissue.^300^ However, proximal tubular-specific knockout DsbA-L mice showed mild renal fibrosis caused by UUO surgery. The mechanism was DsbA-L activated the Hsp90 /Smad3 and p53/CTGF axis.^301^ The relationship between DsbA-L and cGAS-STING pathway under CKD needs further investigation. And the gene expression of cGAS and STING were positively related to renal fibrosis in CKD patients. APOL1 risk allele G2 is associated with high kidney disease risk in African Americans. STING and NLRP3 expression were elevated in podocytes of transgenic mice with APOL1 G2 risk variant and patients with high-risk APOL1 genotypes. Deletion or inhibition of STING and NLRP3 respectively lowered albuminuria and improved kidney function of G2APOL1 mice.^302,303^

A study enrolled 1538 hospitalized patients with renal injury and tested the level of several inflammatory factors in urine. The relationship between inflammatory factors and the progression of CKD was assessed after a median of 4.3 years of follow-up.^304^ The results showed MCP-1/CCL2 and YKL-40 (CHI3L1) levels were negatively associated with eGFR, and uromodulin (UMOD) level was positively associated with eGFR. The renal hostile role of CCL2 and CHI3L1 and the renal protective role of UMOD were also proved in animal studies. This study provided biomarkers of inflammation for renal disease progression. IL23 signaling activated in TECs increased the expression of calcium/calmodulin-dependent protein kinase IV (CaMK4) which suppressed the level of arginine-hydrolyzing enzyme arginase 1 (ARG1), resulting in the release of arginine. Sufficient arginine promoted T cells proliferation and inflammatory cytokines production.^305^ Anti-inflammatory cytokine, IL-10 deficiency aggravated renal fibrosis and inflammation in obesity-related nephrology mice.^306^

### Anti-inflammation therapeutics of CKD

We have reviewed the important signaling pathways and cytokines mediated inflammation in CKD. The targeted drugs are promising for attenuating CKD progression. We introduce drugs in clinical trials first (Table 3).

**Table 3:** The potential drugs undergo clinical trials for the treatment of CKD

Drug	Mechanism	NCT number	Trial information	Primary outcome	Ref.
*Lipotoxicity*					
Simvastatin plus ezetimibe	HMG-CoA inhibitor	NCT00125593	• Phase IV • Enrollment: chronic kidney disease, *n* = 9438	Reduced the risk of atherosclerotic events, nonhemorrhagic stroke, and arterial revascularization	^123^
Atorvastatin	HMG-CoA inhibitor	NCT00296374	• Phase II • Enrollment: diabetic nephropathy, *n* = 353	Modest beneficial effect on eGFR; decreased urine protein: creatinine ratio	^127^
Fenofibrate	PPARα agonist	ISRCTN64783481	• Enrollment: diabetic nephropathy, *n* = 9795	Reduced total cardiovascular events and preserved renal function without adverse effect	^135^
Rosiglitazone	PPARγ agonist	NCT00279045	• Phase III • Enrollment: diabetic nephropathy, *n* = 4426	Decreased ACR compared with metformin, preserved eGFR compared with glyburide	^140^
*Oxidative stress*					
Selonsertib	ASK1 activation	NCT02177786	• Phase II • Enrollment: diabetic nephropathy, *n* = 334	Selonsertib appeared safe, with no dose-dependent adverse effects over 48 weeks. However, mean eGFR for selonsertib and placebo groups did not differ significantly	^207^
Bardoxolone methyl	Nrf2 inducer	NCT00811889	• Phase II • Enrollment: diabetic nephropathy, *n* = 227	Improved the estimated GFR, however, proteinuria and blood pressure were elevated	^204^
Bardoxolone methyl	Nrf2 inducer	NCT01351675	• Phase III • Enrollment: type 2 diabetes mellitus and stage 4 chronic kidney disease, *n* = 2185	The risk of ESRD or death from cardiovascular causes was not reduced even higher in bardoxolone methyl-treated group than in the placebo group	^205^
Bardoxolone methyl	Nrf2 inducer	NCT03019185	• Phase II/III • Enrollment: Alport syndrome, *n* = 187	Change in eGFR from baseline (estimation)	–
Bardoxolone methyl	Nrf2 inducer	NCT03918447	• Phase III • Enrollment: autosomal dominant polycystic kidney, *n* = 550	Change in eGFR from baseline (52 weeks). Count of reported adverse events (104 weeks) (estimation)	–
Pyridorin	Vitamin B6 derivate	NCT02156843	• Phase III • Enrollment: diabetic nephropathy, *n* = 328	Time to composite endpoint of ≥50% or 100% serum creatinine increase from baseline or ESRD (estimation)	–
Pyridoxamine dihydrochloride	Vitamin B6 derivate	NCT00734253	Phase II • Enrollment: diabetic nephropathy, *n* = 317	Pyridorin did not change serum creatinine at 1 year compared with placebo significantly	^200^
*Inflammation*					
Baricitinib	JAK1 and JAK2 inhibition	NCT01683409	• Phase II • Enrollment: diabetic nephropathy, *n* = 129	UACR decreased by 30–40%	^313^
CCX140-B	CCR2 inhibitor	NCT01447147	• Phase II • Enrollment: type 2 diabetic nephropathy, *n* = 332	UACR decreased 16% for 5 mg, 10% for 10 mg compared to placebo	^404^
Canakinumab	IL-1β neutralizing antibody	NCT01327846	• Phase II • Enrollment: CKD patients with stable post-myocardial infarction and high-sensitivity C-reactive protein (hsCRP) ≥2 mg/L, *n* = 10,061	Cardiovascular event rates was reduced 18% on patients with eGFR <60 mL/min/1.73 m^2^ and 16% on patients with eGFR ≥60 mL/min/1.73 m^2^. eGFR, creatinine, and the uACR did not changed by canakinumab	^312^
Canagliflozin	SGLT2 inhibitor	NCT02065791	• Phase III • Enrollment: type 2 diabetes, diabetic nephropathy, *n* = 4401	The relative risk of ESRD in the canagliflozin group was about 30% lower than in the placebo group. Canagliflozin also decreased the risk of cardiovascular events	^310^
Rilonacept	Interleukin-1 receptor antagonist	NCT00897715	• Phase II • Enrollment: chronic kidney disease, *n* = 15	Concentration of high-sensitivity C-reactive protein (hsCRP) decreased by 54%	–
*Myofibroblasts activation and ECM production*					
VPI-2690B	Monoclonal antibody to αvβ3 integrin	NCT02251067	• Phase II • Enrollment: diabetic nephropathy, *n* = 165	Albuminuria reduction and eGFR preservation during the 50-week trial duration (estimation)	^199^
Pirfenidone	Decreased TGF-β synthesis	NCT00063583	• Phase I, II • Enrollment: diabetic nephropathy, *n* = 52	The mean eGFR increased in the pirfenidone 1200 mg/d group, whereas the mean eGFR decreased in the placebo group	^357^
Pirfenidone	Decreased TGF-β synthesis	NCT00001959	• Phase II • Enrollment: FSGS, *n* = 21	GFR decreased during baseline period and treatment period was the same	–
Pirfenidone	Decreased TGF-β synthesis	NCT02408744	• Phase I, II • Enrollment: CKD, *n* = 30	The progression of renal damage in patients with CKD was evaluated	–
Pirfenidone	Decreased TGF-β synthesis	NCT02408744	• Phase II • Enrollment: CKD, *n* = 200	Change from baseline in kidney fibrosis	–
LY2382770	TGF-β1 neutralizing monoclonal antibody	NCT01113801	• Phase II • Enrollment: diabetic nephropathy, *n* = 417	Failed to change in serum creatinine from baseline to 12-month endpoint	–
Fresolimumab	TGF-β neutralizing monoclonal antibody	NCT01665391	• Phase II • Enrollment: SR-FSGS, *n* = 88	Fresolimumab (1 mg/kg) was well tolerated, and decreased urinary protein/creatinine ratio at day 112 compared to the placebo group	^358^
Finerenone	Steroidal mineralocorticoid receptor antagonist	NCT01874431	• Phase II • Enrollment: diabetic nephropathy with high albuminuria, *n* = 823	Finerenone decreased UACR in patients with diabetic nephropathy receiving ACEis or ARBs in a dose-dependent way	^398^
Finerenone	Steroidal mineralocorticoid receptor antagonist	NCT02540993	• Phase III • Enrollment: CKD, *n* = 5734	17.8% patients in the finerenone group occurred sustained decrease of at least 40% in the eGFR and 21.1% in the placebo group	^399^

Sodium-glucose cotransporter 2 (SGLT2) inhibitors inhibit glucose reabsorption by targeting SGLT2 proteins which are mainly expressed by proximal TECs. The mechanisms for the renoprotective effect of SGLT2 inhibitor are not only due to glycemic control, but also inflammation and OS depression. The NLRP3 inflammasome activation and ATP-induced secretion of IL-1β were inhibited in the macrophages isolated in type 2 diabetes mellitus (T2DM) patients by SGLT2 inhibitor, empagliflozin treatment.^307^ Empagliflozin inhibited the renal expression of MCP‐1, TGF‐β, NF‐κB, and IL‐6, and circulating levels of MCP‐1, IL‐6, and TNF‐α in DN animals.^308^ Transcriptomic analysis of plasma biomarkers showed canagliflozin reduced the mRNA level of TNF receptor 1, IL-6, and matrix metalloproteinase 7 (MMP7) in patients with T2DM compared with glimepiride treated group.^309^ A phase 3 trial (NCT02065791) results showed the relative risk of ESRD in the canagliflozin group was 30% lower than the placebo group at a median follow-up of 2.62 years.^310^ Canagliflozin also decreased the risk of cardiovascular events in patients with DN. Based on the positive results, further clinical trials about SGLT2 inhibitors were conducted. The effect of empagliflozin on proteinuria and kidney disease progression in patients with non-diabetic glomerulonephritis is under investigation (Phase 3 clinical trial, NCT05283057). And the recruitment is completed, but the results are not reported yet. A phase 4 clinical trial (NCT05225077) that evaluates the effect of short-term dapagliflozin on renal function after heart catheterization or percutaneous coronary intervention in patients with CKD is recruited. Exciting clinical trial results provide strong evidence for the use of SGLT2 inhibitors in patients with DN, even in CKD patients without diabetes. A phase 2 trial (RESCUE, NCT03926117) evaluated the effect of IL-6 inhibitor, ziltivekimab on the percentage change of high-sensitivity CRP in adult patients with moderate to severe CKD and high-sensitivity CRP of at least 2 mg/L.^311^ After 12 weeks, ziltivekimab reduced the levels of high-sensitivity CRP significantly in a dose-dependent way. In the highest 30 mg dosage group, high-sensitivity CRP decreased by 92% compared with 4% for the placebo group. It did not affect the total cholesterol to HDL-cholesterol ratio and was well tolerated. The conclusion of the RESCUE trial provided a promising drug for lowing the risk of atherosclerosis in CKD patients by inhibiting inflammation. And further studies are needed to assess whether ziltivekimab attenuates CKD progression and reduced the risk of cardiovascular events. The renal protective role of IL-6 inhibitor has been demonstrated in animal experiments. Experiments in animal models showed IL-6 specific inhibitor, Fc-gp130 attenuated renal fibrosis and immune cells infiltration.^259^ The reducing cardiovascular event rates and renal progression effects of canakinumab, a human monoclonal antibody neutralizing IL-1β, were evaluated on CKD patients with stable post-myocardial infarction and high-sensitivity C-reactive protein ≥2 mg/L. The phase 2 clinical trial (NCT01327846) results showed canakinumab reduced major adverse cardiovascular event rates compared with placebo treatment over a median follow-up period of 3.7 years. However, renal function reflected by eGFR, creatinine, and the urine ACR were not influenced by canakinumab.^312^ The effect on decreasing albuminuria and inflammatory biomarkers of JAK1/2 inhibitor, baricitinib, was evaluated in elder patients with diabetic kidney disease (NCT01683409). After 24 weeks of treatment, baricitinib decreased morning ACR by 41% compared with placebo. And the level of uric inflammatory biomarkers including CXCL10, CCL2, soluble TNFR1 and 2, SAA, and cell adhesion molecule 1 was decreased by barcitinib. However, about 10 folds of anemia occurred in the baricitinib group compared with the placebo. The conclusion was not so convincing due to it was drawn from the results of less than 30 patients.^313^ Further study is needed to evaluate the safety of barcitinib. Multicenter preclinical randomized controlled trials (pRCT) evaluated the effect of baricitinib on lupus nephritis. MRL/MpJ-Faslpr mice were randomized to oral administration with baricitinib or vehicle at four dependent centers. Data analysis was performed at an independent fifth site. The results showed baricitinib did not decrease proteinuria and renal injury.^314^ pRCT predicted patients with lupus nephritis would not benefit from baricitinib.

Except for drugs in clinical trials discussed above, a large number of novel CKD treatments that inhibit inflammation have been tested on animal models. NF-κB is no doubt an important target. Arctigenin (ATG) reduced proteinuria in diabetic patients and renal fibrosis of UUO mice by inhibiting the NF-κB pathway in phosphatase 2A (PP2A) dependent way. PP2A also dephosphorylated the actin cytoskeleton-related proteins Drebrin-1 (DBN1) on the T335 site, and then increased adhesion and reduced the migration of podocytes.^315^ An NF-κB inhibitor, pyrrolidine dithiocarbamate, was revealed to attenuate renal inflammation and fibrosis in multiple CKD animal models, such as gentamicin-treated rats, 5/6 nephrectomy rats, and aldosterone and salt-induced renal disease.^229^ Zinc supplementation ameliorated vascular calcification of CKD by depressing the NF-κB pathway.^316^ The possible mechanism was that Zinc bound to zinc-sensing receptor ZnR/GPR39 and upregulated the expression of zinc-finger protein TNF-α-induced protein 3 (TNFAIP3, also known as A20) which is a suppressor of NF-κB. The major stimulus of NF-κB, TNF-α, can be inhibited by infliximab or etanercept. The TNF-α inhibition decreased proteinuria and attenuated CKD progression in various CKD animal models. Adalimumab which was a neutralizing antibody of TNFα was applied to patients with primary focal segmental glomerulosclerosis (FSGS) in the FONT trial (NCT00814255).^317^ However, the trial ultimately ended due to under-enrollment. A case report showed 33-year-old man treated with adalimumab for 7 years appeared with renal dysfunction, nephrotic proteinuria, and erythrocyte uria. The albuminuria and renal function were back to normal when adalimumab was abandoned.^318^ And similar case reports were described.^319,320^ The effects and safety of adalimumab should be evaluated in further study. PLK1 inhibitor (BI-2536) was predicted to be the top candidate for depressing 15 genes expression related to DN with a connectivity mapping approach and Integrated Network-based Cellular Signatures (LINCS) L1000 data set querying.^321^ Indeed BI-2536 ameliorated proteinuria and kidney injury of DN mice by dampening NF-κB and Smad3 signaling.

The inhibitors of JAK-STAT signaling targeting each process including receptors, JAK activation, STAT activation, and STAT binding to DNA have been explored extensively.^322^ Here, we list some inhibitors that have been tested on CKD. Nifuroxazide was found to inhibit the activation of STAT3 with the STAT3-dependent luciferase reporter cell line. Further research identified STAT1, STAT5 and JAK2 also can be blocked by nifuroxazide.^323^ Nifuroxazide treatment significantly reduced renal inflammation and improved glomerular filtration function and diabetic kidney disease-related renal structural alterations.^324^ Similar results were observed in UUO mice treated with nifuroxazide.^325^ Small molecule S3I-201 and Stattic protected kidneys from injury in multiple animal models with CKD by inhibiting the SH2 domain of STAT3.^322^ JAK1/2 inhibitor, ruxolitinib, protected podocytes from high glucose injury.^326^ JAK2/3 inhibitor, AG490 reduced inflammation, tubular apoptosis, and interstitial fibrosis of UUO mice and proteinuria of DN mice.^199,327^

MCC950 inhibited the NLRP3 activation inflammasome formation by binding to the nucleotide-binding and oligomerization domain and altering the protein structure of NLRP3.^328,329^ MCC950 reduced NLRP3 inflammasome activation and the following caspase 1 activation and IL-1β release in podocytes. Thus, MCC950 attenuated proteinuria and renal lesions of mice with lupus nephritis.^330^ Luteolin, a natural flavonoid compound, prevented podocyte injury from high glucose injury by inhibiting NLRP3 inflammasome activation.^331^ Xenon, an inert anesthetic gas decreased serum levels of anti-double-stranded DNA autoantibody and neutrophil chemokines, thereby improving kidney function and renal histology by blunting NF-κB/NLRP3 inflammasome activation.^332^ IL-6 receptor inhibitor, tocilizumab, suppressed inflammatory response and OS of kidney in diabetic mice.^333^ β-hydroxybutyrate precursor, 1,3-butanediol, protected mice from nephrocalcinosis-related CKD by inhibiting NLRP3 inflammasome activation.^334^ The similar renoprotective effect of β-hydroxybutyrate was revealed by another study. The results showed that β-hydroxybutyrate administration decreased the expression of caspase 1 and pyroptosis of proximal tubular cells after ischemia-reperfusion injury.^335^ Anakinra, a recombinant interleukin-1 receptor antagonist attenuated kidney progression in mice with DN and IgA nephropathy, but not in lupus nephritis.^336^ Caspase inhibitor, M-920 inhibited caspase 1-dependent inflammasome activation and then ameliorated DN.^337^

HSP70-TLR4 axis contributed to the albuminuria, renal inflammation, and progression of STZ-induced diabetic mice, and HSP70 inhibitors, VER-155008 and pifithrin-μ protected the kidney from injury.^338^ TLR4 antagonist, CRX-526, inhibited the upregulation of osteopontin which mediated the crosstalk between TECs and fibroblasts and negatively correlated with kidney function, and NF-κB nuclear translocation, thus significantly reducing albuminuria and renal injury.^339,340^ TAK-242 was another inhibitor of TLR4 and proved to inhibit M2 macrophage polarization and renal inflammation after UUO surgery.^341^

We have summarized the effects and regulation of core inflammatory signaling pathways and the targeted drugs tested in clinical trials and animal models of CKD. Several anti-inflammatory drugs perform exhilaratingly in preventing CKD progression, reducing proteinuria or the risk of cardiovascular disease. And further clinical trials are needed to specify the application of these drugs. As we discussed above, various anti-inflammatory inhibitors are investigated on animal models of CKD and show satisfactory results. The possible reason could be inflammatory signaling pathways are crosslinking with each other. Thus, one process inhibition arises amplified dominoes. However, this is also the reason for the unsatisfactory effects of some inhibitors due to the compensation from another signaling. Finding the key factors that control inflammation is important for discovering effective anti-inflammatory inhibitors.

## Key pathways mediated myofibroblasts activation and ECM deposition

**Fig. 6:**
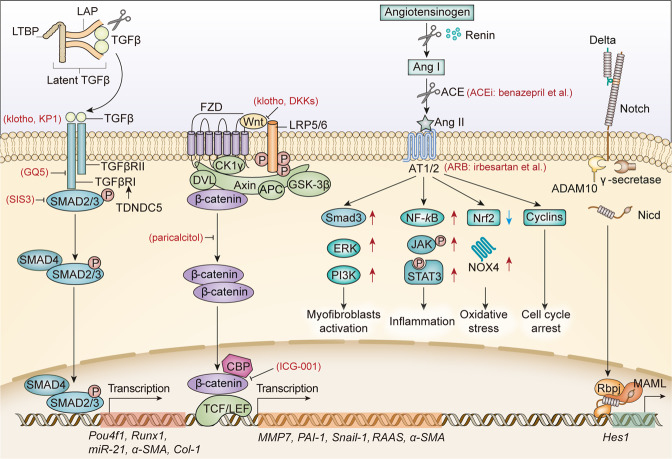
Key pathways mediated myofibroblasts activation and ECM deposition. In TGF-β signaling, TGF-β1 released from LTBP binds with TGFBR2, which recruits TGFBR1, inducing the downstream phosphorylation of Smad2 and Smad3. P-Smad2 and P-Smad3 form a complex with Smad4 and translocate into the nucleus, promoting the transcription of profibrotic genes and inhibiting the antifibrotic factors. In activated Wnt/β-catenin signaling, Wnt ligands bind with Frizzled receptor and co-receptor LRP5/6 to inhibit the assembling of the β-catenin destruction complex. Cytoplasm accumulated β-catenin translocates into the nuclear and binds to TCF/LEF to promote downstream target genes transcription. Notch signaling is triggered by the interaction between ligands and receptors, inducing the cleavage by ADAM protease and γ-secretase complex. The product NICD is transported into the nucleus to regulate the transcription of downstream genes with the assistance of Rbpj and MAML. The cascade of RAAS is that renin converts angiotensinogen to angiotensin I. Under the action of ACE, Ang I is converted into Ang II, which can bind to AT1R to activate multiple pathways such as NF-κB signaling. Red fonts in brackets are inhibitors. TGF-β transforming growth factor-beta, LTBP latent TGF-β binding protein, TGFBR1 TGF-β receptor 1, TGFBR2 TGF-β receptor 2, TCF T-cell factor, LEF lymphoid enhancer-binding factor, LRP low-density lipoprotein-related protein, NICD Notch intracellular domain, MAML Mastermind-like Protein, RAAS renin-angiotensin-aldosterone system, Ang I angiotensin I, ACE angiotensin-converting enzyme, AT1R angiotensin II receptor type 1

Myofibroblasts are regarded as the key producers of ECM. The capacity for generating collagen fibers of myofibroblasts is much greater than fibroblasts. α-smooth muscle actin (α-SMA) is a classical but not specific marker of myofibroblasts. Platelet-derived growth factor receptor β, fibroblast-specific protein 1, CD73, and vimentin are also non-specific markers of myofibroblasts.^342^ Thus, more than two markers are used to identify myofibroblasts usually. With the development of signal-cell RNA sequencing technology, more markers such as naked cuticle homolog 2 were discovered.^212^ Myofibroblasts are hard to detect in healthy kidneys but increase greatly in the fibrotic kidney. Proliferation and activation are two pathways to obtain numerous myofibroblasts. Renal resident fibroblasts, pericytes, TECs, and endothelial cells, and bone-marrow-derived cells such as macrophages, mesenchymal stem cells were reported to be the precursors of myofibroblasts.^343,344^ Developmental pathways including TGF-β signaling, Wnt/β-catenin pathway, Notch signaling, and RAAS signaling play an important role in the activation and proliferation of myofibroblasts and the production of ECM (Fig. 6).

### TGF-β signaling

TGF-β, a member of the transforming growth factor superfamily consists of three isoforms including TGF-β1, TGF-β2, and TGF-β3. TGF-β1 is the most well-studied isoform in renal fibrosis. TGF-β1 is synthesized as a precursor form. And the precursor of TGF-β1 interacts with latency-associated peptide and latent TGF-β-binding protein to form the complex that keeps TGF-β1 in an inactive state.^345^ Overexpression of latent TGF-β reduced the expression of active TGF-β1 and ECM-related proteins such as collagens after UUO injury compared to wild-type mice.^346^ However, the complex is cleaved in the injured kidney by proteases including plasmin, MMP2 and MMP9, and thrombospondin to release active TGF-β1.^347,348^ The active TGF-β1 triggers classical and nonclassical pathways. The classical TGF-β cascade is TGF-β1 binds to TGFBR2 directly and phosphorylates TGFBR2. And the phosphorylated TGFBR2 recruits and activates TGFBR1. The active TGFBR1 then phosphorylates Smad2 and Smad3. Phosphorylated Smad2 and Smad3 translocate into the nucleus with the assistance of Smad4. The phosphorylated Smad3 directly binds to the promoter of targeted genes to modify the transcription.^349^ The transcription of Smad7 is also increased by phosphorylated Smad3. And Smad7 acts as a negative regulator of the TGF-β/Smad3 pathway by competing with Smad2 and Smad3 for the binding sites on activated TGFBR1.^350^ The binding of TGF-β1 and TGFBR2 also triggers Smad independent pathways including JNK, MAPK, and ERK.^349^

The expression of components of TGF-β signaling is upregulated in fibrotic kidneys of patients and animals with CKD. Classical and nonclassical pathways of TGF-β are activated to promote the activation and proliferation of myofibroblasts, and the generation of ECM.^349^ Phosphorylated Smad3 induced by TGF-β directly regulates the expression of α-SMA which is the classical marker of myofibroblasts, and ECM proteins such as Collagens, fibronectin.^351^ The upregulation of TGF-β breaks the balance between ECM synthesis and degradation. TGF-β1 treatment inhibited the expression of PAs and induced the production of their inhibitor PAI-1, leading to the reduced degradation of ECM.^352^ Tissue inhibitor of metalloproteinase that was an inhibitor of MMP was upregulated by TGF-β/Smad signaling in CKD and promoted age-related renal fibrosis.^353^ Precursors like TECs, endothelial cells, and macrophages convert into myofibroblasts through partial epithelial-mesenchymal transition (partial EMT), endothelial-mesenchymal transition (EndMT), and MMT, respectively. Runt-related transcription factor 1 (RUNX1) mediated partial EMT in a Smad3-dependent way.^354^ Brain-specific homeobox/POU domain protein Pou4f1 (Brn3a) is also the target of Smad3. TGF-β/Smad3 upregulated the expression of Pou4f1 which was a key mediator of MMT.^214^ The upregulated thioredoxin domain containing 5 (TXNDC5) enforced TGF-β signaling activity and promoted fibroblasts activation by post-translational stabilizing and upregulating TGFBR1.^355^ TGF-β/Smad3 signaling also regulates the expression of multiple noncoding RNAs such as miR-21 which is a well-known profibrotic miRNA.^351^ miR-21 inhibitor, lademirsen (SAR339375), has been conducted in phase 2 clinical trial (NCT02855268) which assesses the efficacy in reducing the decline in renal function of patients with Alport syndrome. Long noncoding RNA (lnc-TSI) blocked the interaction of Smad3 with TGFBR1 by binding with the MH2 domain of Smad3 and attenuated renal fibrosis.^356^

Due to the strong profibrotic effects, TGF-β signaling has been considered to be a targeted pathway to attenuate renal fibrosis. And the therapies that targeted TGF-β signaling have been widely studied in the past few decades. Pirfenidone is a synthetic small molecule inhibiting TGF-β synthesis. A phase 2 clinical trial (NCT00001959) of pirfenidone in patients with FSGS showed eGFR decreased during the baseline period and pirfenidone treatment inhibited the eGFR decline. Another clinical trial showed pirfenidone elevated the mean eGFR compared with placebo in patients with DN.^357^ The positive results of these clinical trials make pirfenidone a promising drug for attenuating the progression of CKD. Fresolimumab, a monoclonal anti-TGF-β antibody was applied to patients with steroid-resistant focal segmental glomerulosclerosis (SR-FSGS). Although the clinical trial (NCT01665391) did not meet the endpoint, at day 112, the mean percent of urinary protein/creatinine ratio decreased significantly in patients treated with fresolimumab 1 mg/kg.^358^ However, TGF-β1-specific, humanized, neutralizing monoclonal antibody (TGF-β1 mAb) did not decrease SCr compared to placebo in patients with DN.^359^ And similar results were concluded from a phase 2 clinical trial of LY2382770 (NCT01113801). Limitations of the trial confounded drawing definitive conclusions that TGF-β had no role in the pathogenesis of DN. And another possible reason is other pathways are also important for the progression of renal fibrosis. Bardoxolone, an Nrf2 activator, protected the kidney from injury by increasing Smad7 levels and inhibiting TGF-β/Smad expression.^360^ The novel inhibitors of TGF-β signaling are under investigation. Klotho was an anti-aging protein that was discovered in 1977 by Japanese scientists.^361^ Secreted α-Klotho inhibited TGF-β signaling by directly binding to TGFBR2 and ameliorated renal fibrosis.^362^ However, recombinant full-length Klotho supplement is difficult to apply to CKD patients due to the high cost and difficulties to produce active recombinant full-length Klotho. Liu et al. found the core sequence of α-Klotho for binding to TGFBR2 and inhibited the activation of classical and nonclassical TGF-β signaling. The Klotho-derived peptide, KP1, mimicked the renal-protected function of full-length Klotho and ameliorated renal fibrosis of UUO and UIRI mice.^363^ SIS3 is a specific inhibitor abolishing Smad3 but not Smad2 phosphorylation and attenuates renal fibrosis in diabetic and obstructive nephropathy.^364^ GQ5 inhibited the phosphorylation of Smad3 by blocking the interaction of Smad3 with TGFBR1 and attenuated fibrotic lesions in obstructive nephropathy.^365^

### Wnt/β-catenin signaling

The Wnt/β-catenin pathway is evolutionarily conserved and regulates embryonic development, carcinogenesis, and tissue injury and repair.^366^ Wnt proteins are a conserved family of secreted lipid-modified glycoproteins, which includes 19 different members in mammalian species: Wnt1, 2, 2b, 3, 3a, 4, 5a, 5b, 6, 7a, 7b, 8a, 8b, 9a, 9b, 10a, 10b, 11, and 16. As a type of glycolipoprotein, Wnt proteins are modified before secreting. The N-terminal cysteine-rich residues of Wnts are glycosylated in the ER and the C-terminal serine 209 residue is palmitoylated by a membrane-bound acyltransferase, known as porcupine.^367^ Transport of the lipid-modified Wnt ligands is regulated by Wntless (Wls). After being released from cargo receptor, extracellular Wnt ligands bind to plasma membrane receptors, Frizzled receptor family of proteins, and co-receptors, low-density lipoprotein-related protein 5 and 6 (LRP5/6). The (pro)renin receptor is necessary for the activation of Wnt signaling.^368^ Then the signal transmits to cytoplasmic phosphoprotein, disheveled (Dsh/Dvl). Wnt ligands activate the canonical, β-catenin-dependent pathway, and the noncanonical, β-catenin-independent pathway. In a healthy kidney, Wnt signaling is in an inactive state. β-catenin is constitutively phosphorylated by casein kinase 1 (CK1) and glycogen synthase kinase-3β (GSK-3β), and degraded by ubiquitin-mediated proteolysis in the E3 ubiquitin ligase β-TrCP dependent way.^369^ However, the upregulated Wnt ligands in fibrotic kidney bind to Frizzled and LRP5/6 and inhibit the binding of APC, CK1, GSK-3β, and β-catenin to the scaffold protein, Axin1. The destruction complex of β-catenin collapses. Cytoplasm accumulated β-catenin translocates into the nuclear and binds to T-cell factor/lymphoid enhancer-binding factor to promote downstream target genes transcription.^370^

Wnt/β-catenin signaling is important signaling mediated renal fibrosis. Wnt/β-catenin signaling is activated in AKI and CKD. The increased Wnt ligands and β-catenin promoted renal repair in AKI. However, the sustained activation of Wnt/β-catenin signaling aggravated renal fibrosis.^371^ Loss of α-Klotho amplified Wnt/β-catenin signaling in patients and animals with CKD.^372^ ECM proteins such as fibronectin, partial EMT driver Snail-1, ECM degradation mediators MMP7 and PAI-1, and all the components of the RAAS are downstream targets of β-catenin.^373,374^ Wnt3a exacerbated IL-4- or TGF-β1-induced macrophage alternative M2 polarization. Deletion of β-catenin in macrophages attenuated the ECM deposition and renal inflammation by limiting M2 macrophage polarization.^375^ Overexpression of Wnt9a drove tubular senescence and produce TGF-β1, which promoted proliferation and activation in normal rat kidney fibroblasts.^376^ Tubule-derived Wnts but not fibroblasts-derived Wnts were necessary for fibroblasts activation and renal fibrosis.^377^ Moreover, β-catenin expressed by TECs controlled the release of osteopontin (OPN) enriched exosome which promoted fibroblast proliferation and activation by binding to CD44.^340^

Blockade of Wnt/β-catenin signaling is a potential therapy for ameliorating renal fibrosis. α-Klotho abolished the interaction between Wnt ligands and the receptors and then inhibited the activation of Wnt/β-catenin signaling. Secreted α-Klotho supplement suppressed myofibroblast activation, reduced matrix expression, and ameliorated renal fibrosis.^372^ Paricalcitol hampered activation of renal myofibroblasts and suppressed expression of the fibrogenic factors. Mechanistically, paricalcitol blunted β-catenin-mediated gene transcription by inducing the interaction between vitamin D receptors and β-catenin.^378^ Small molecule ICG-001 inhibited the binding of β-catenin and CREB binding protein (CBP) and abolished β-catenin-driven gene transcription, resulting in the attenuated fibrotic lesions in obstructive nephropathy.^379^ Dickkopf (DKK) families including DKK1-4 are natural antagonists of Wnt/β-catenin signaling by binding to LRP6 and blocking the interaction between Wnt ligands and LRP6.^380^ Moreover, DKK1 and DKK2 induce endocytosis of LRP6. Various studies showed DKKs reduced myofibroblasts activation and counteracted kidney fibrosis.^373,381^ A phase 2 clinical trial (NCT01337752) evaluating the effect of anti- DKK1 monoclonal antibody (BHQ880) compared with placebo on time to first skeletal-related event in patients with untreated multiple myeloma and renal insufficiency in combination with bortezomib and dexamethasone is recruiting. However, DKK1 promotes mesangial matrix accumulation and proteinuria in mice with DN.^382^ The effects of DKKs may differ from diseases. Although the renal protective role of Wnt/β-catenin signaling inhibition has been proved in multiple CKD animal models, there are no clinical trials of drugs targeting Wnt/β-catenin signaling in patients with CKD currently.

### Notch signaling

The Notch pathway is a phylogenetically conserved pathway, which controls the signal communication between adjacent cells. The ligands of Notch signaling, Jagged 1, 2, and Delta 1, 3, 4 are expressed on the cell membranes. In the interaction of the ligands with the receptors, Notch 1-4 initiates the successive cleavage of the receptors mediated by ADAM protease and γ-secretase complex. Finally, the Notch intracellular domain is transported to the nucleus and regulates the transcription of downstream genes with the assistance of transcriptional regulators, such as Rbpj and MAML.^383^

Both ligands and receptors of Notch signaling are upregulated in the injured kidney of patients and animals with DN, FSGS, minimal change disease, and lupus nephritis, and of animals with folic acid nephropathy, puromycin aminonucleoside-induced nephropathy, and UUO.^384^ The degree of glomerulosclerosis was positively related to the levels of cleaved Notch1 expressed by podocytes, while the severity of tubulointerstitial fibrosis and the estimated GFR were positively related to the levels of cleaved Notch1 in the tubulointerstitium.^385^ Tubular overexpressing cleaved Notch1 genetic mice aggravated renal fibrosis induced by folic acid. However, tubular-specific deletion of Rbpj, an important transcriptional regulator of Notch signaling, reduced the transcription of ECM proteins, and myofibroblasts markers including vimentin and α-SMA.^386^ Notch3 is also a critical isoform involved in kidney fibrosis. Notch3 deficiency protected UUO mice from tubular damage and cell loss with significantly reduced interstitial collagen deposition.^387^ Loss of histone deacetylase Sirt6 increased the transcription of Notch1 and Notch4 of podocytes.^388^ The activation of Notch signaling exacerbated podocyte injury, proteinuria, and glomerulosclerosis in mice with DN and adriamycin-induced nephropathy. Aberrant activation of Notch1 signaling in glomerular endothelium induced severe albuminuria which was an independent risk factor of glomerulosclerosis.^389^

We have introduced the critical role of Notch signaling in promoting the progression of CKD. Notch signaling inhibition is a probable solution for CKD. γ-secretase inhibitor DBZ treatment diminished the transcript levels of fibrosis markers, including collagen 1a1, collagen 3a1, fibronectin, and decreased the activation of myofibroblasts reflected by α-SMA and vimentin in mouse models of folic acid and UUO- induced kidney disease.^386^

### The activation of RAAS

RAAS is important for regulating blood pressure homeostasis, vascular injury, and repair responses. Renin is mainly produced by renal juxtaglomerular cells, and in circulation, renin converts liver-derived angiotensinogen to angiotensin I (Ang I). Under the action of ACE, Ang I is converted into the biologically active Ang II, which can bind to angiotensin II receptor type 1 (AT1) to initiate the downstream cascade reaction.^390,391^ There is no classical pathway that mediated the signal transduction after Ang II binding to AT1R. Traditionally recognition is RAAS activated in circulation due to the components being produced in different organs. However, local RAAS activation evokes attention. All the components of RAAS are upregulated in the fibrotic kidney. The activation of AT1 triggers multiple signaling pathways, for example, kinase activation including ERK, PI3K, AKT, and JAK-STAT, activation of transcription factors including Smad, NF-κB, and Nrf2, OS, and cell cycle-related factors.^392^ The activation of RAAS promotes the progression of renal fibrosis through various pathways. RAAS also mediates blood pressure elevation and cardiac fibrosis.^391,393^

ACEis and ARBs are the prime therapies for CKD patients. The CAPTOPRIL trial showed ACEi, captopril, reduced the proteinuria of patients with DN and proteinuria by 30%; decreased the risk of double serum creatine levels by 43%; decreased the risk of death or dialysis or renal transplantation by 50% compared to placebo.^394^ The exciting results drove ACEi to an effective and classical drug for CKD patients with serum creatine less than 2.5 mg/dL. An impressive clinical trial expanded the range of ACEis applied to CKD patients. The results showed ACEi, benazepril, reduced the risk of doubling the serum creatinine level, ESRD, or death by 43% compared to placebo in CKD patients with serum creatinine levels of 3.1 to 5.0 mg/dL.^395^ Although ACEis are effective and widely used, some patients would have the side effects such as cough. And ARBs such as irbesartan can be alternative drugs. The physiological effect of aldosterone is controlling sodium reabsorption and potassium secretion through binding to the mineralocorticoid receptor (MR). The activation of MR in CKD leads to OS, inflammation, fibrotic remodeling, and hemodynamic alterations.^396,397^ Finerenone is a nonsteroidal, selective MR antagonist. A clinical trial conducted in 2015 demonstrated finerenone decreased the urinary ACR in patients with DN receiving ACEis or ARBs in a dose-dependent way.^398^ A further study evaluated the long-term outcome of finerenone on patients with CKD and type 2 diabetes. The results showed that 17.8% of patients in the finerenone group occurred a sustained decrease of at least 40% in the eGFR and the ratio was 21.1% in the placebo group. 13.0% of patients in the finerenone group occurred cardiovascular events, and 14.8% in the placebo group.^399^ And the *P* values were both lower than 0.05. It was concluded that finerenone lowered the risks of CKD progression and cardiovascular events in patients with DN.

## Conclusions

Lipotoxicity, OS, inflammation, and fibrosis play an important role in promoting the progression of CKD. We have discussed the key signaling pathways that regulated these primary pathologic changes and the therapies targeted to these pathways. The ectopic lipid and lipoprotein metabolism not only is the result of CKD but also accelerates CKD progression. Lowering lipid therapies such as stains preserve renal function and decrease proteinuria and cardiovascular events for CKD patients. However, the available lipid-lowering drugs are limited, especially for CKD patients undergoing dialysis. The possible reason might be that, although the serum lipid levels are controlled, the genetic disorders of the kidney cannot be rectified easily. The signaling pathways mediated OS and inflammation form a complex network. The generation of ROS activates inflammatory factors such as NLRP3 inflammasome and NF-κB. And the reinforced inflammation enhances the production of ROS. Anti-inflammation drugs and anti-OS drugs get great achievements for CKD treatment recently. SGLT2 inhibitors are novel available drugs for preventing the decline of renal function. Anti-fibrosis drugs perform satisfying in CKD animals and patients. ACEi and ARB are classical drugs for CKD treatment. Inhibitors targeting TGF-β signaling are promising drugs for CKD patients. However, the signaling pathways of fibrosis have been studied for several decades, and targeted drugs in clinical trials are limited. Further investigations on the signaling pathways that regulated CKD and the targeted therapies are needed.
